# Supplementary material for: Remodeling of Bone Marrow Hematopoietic Stem Cell Niches Promotes Myeloid Cell Expansion during Premature or Physiological Aging
Source: Cell Stem Cell. 2019 Sep 5;25(3):407–418.e6. doi: 10.1016/j.stem.2019.06.007 (PMC6739444; doi:10.1016/j.stem.2019.06.007)
Supplement: Document S1. Figures S1–S7 [file mmc1.pdf]

Cell Stem Cell, Volume *25*

Supplemental Information

Remodeling of Bone Marrow Hematopoietic Stem Cell Niches Promotes Myeloid Cell Expansion during Premature or Physiological Aging

Ya-Hsuan Ho, Raquel del Toro, José Rivera-Torres, Justyna Rak, Claudia Korn, Andrés García-García, David Macías, Cristina González-Gómez, Alberto del Monte, Monika Wittner, Amie K. Waller, Holly R. Foster, Carlos López-Otín, Randall S. Johnson, Claus Nerlov, Cedric Ghevaert, William Vainchenker, Fawzia Louache, Vicente Andrés, and Simón Méndez-Ferrer

Figure S1, Related to Figure 1 and Figure 2

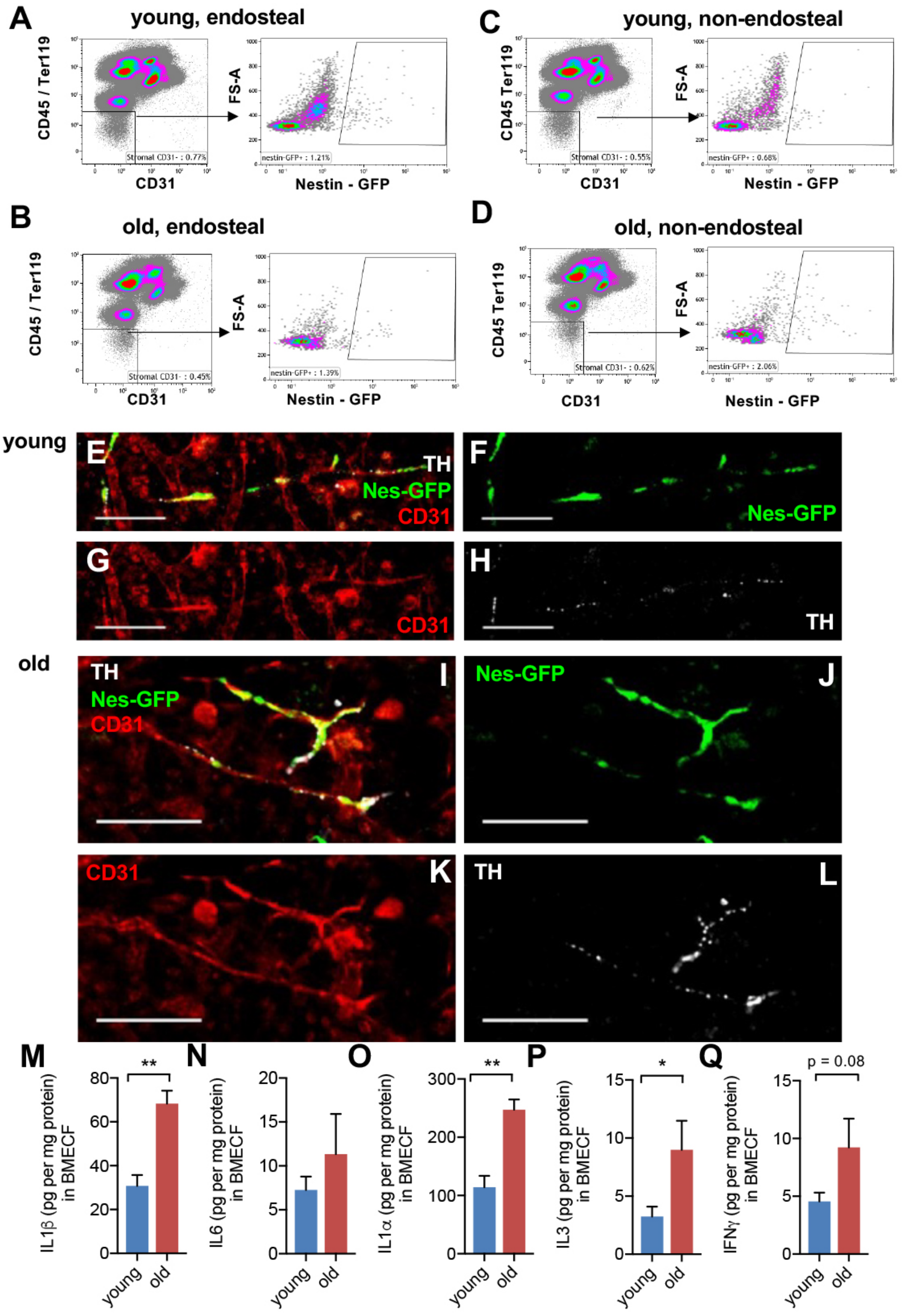

Figure S1, Related to Figure 1 and Figure 2. Reduction of endosteal niches and expansion of non-endosteal neurovascular niches correlates with increased myelopoietic cytokines during BM aging. (A-D) Representative flow cytometry diagrams from BM $CD45^-$ $Ter119^-$ $Nes\text{-}GFP^+$ cells isolated from (A, B) endosteal and (C, D) non-endosteal BM from (A, C) young and (B, D) old *Nes-gfp* mice. The frequencies of gated population are indicated. (E-L) Additional examples showing the immunofluorescence of tyrosine hydroxylase $(TH)^+$ sympathetic nerve fibers (white), $CD31^+$ endothelial cells (red), and GFP^+ cells (green) in the tibial BM of (E-H) young and (I-L) old *Nes-gfp* mice. Scale bar, 100 µm. (M-Q) Protein concentration of (M) IL1β, (N) IL6, (O) IL1α, (P) IL3 and (Q) IFNγ in non-endosteal BM extracellular fluid (BMECF) from young WT mice ($n = 3$) and old WT mice ($n = 7$). Data are means ± SEM. * $p < 0.05$; ** $p < 0.01$. Unpaired two-tailed t test.

Figure S2, Related to Figure 3

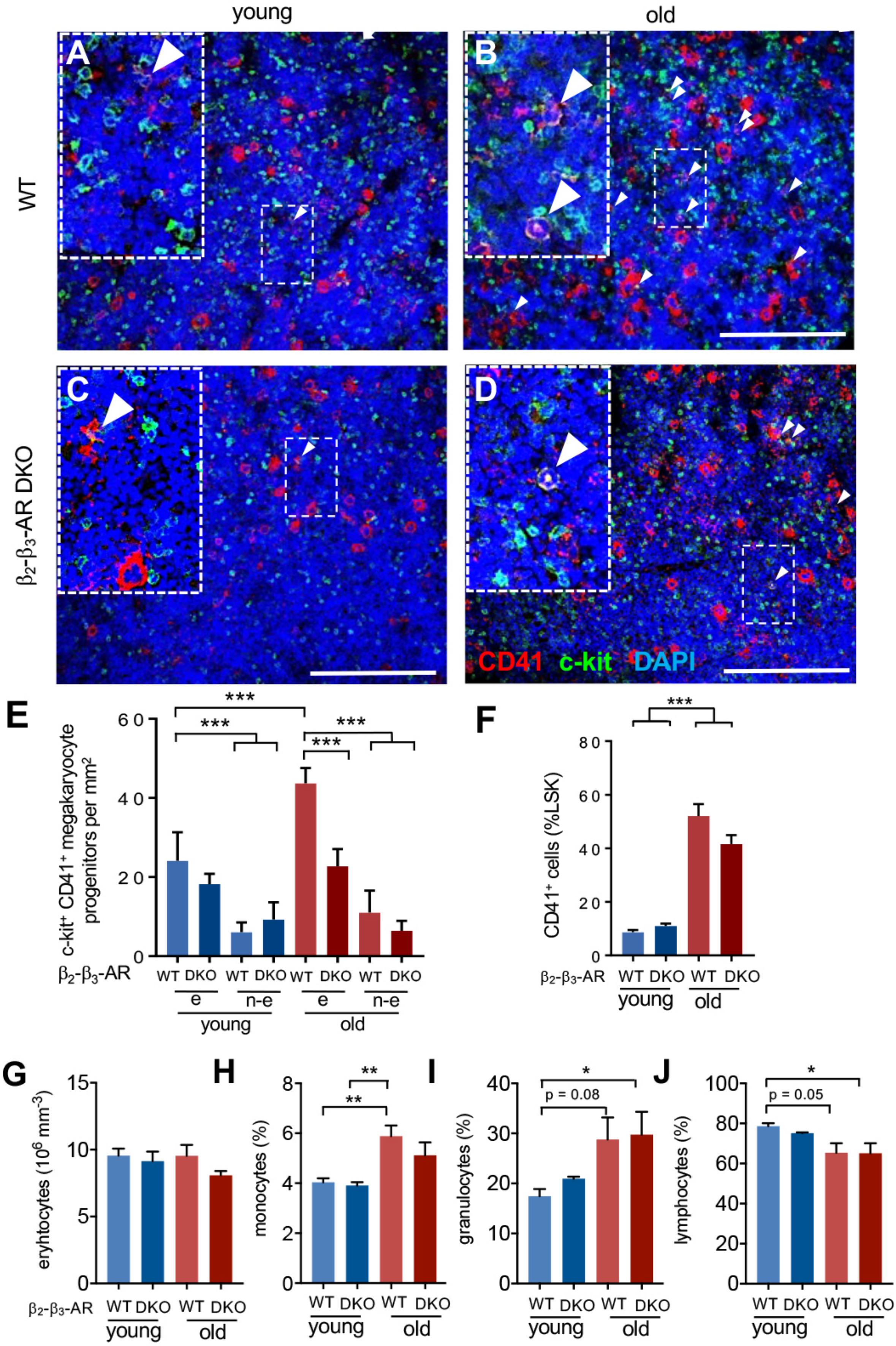

Figure S2, Related to Figure 3. β-adrenergic signals promote megakaryopoiesis during aging. (A-D) Representative immunofluorescence staining for CD41 (red) and c-kit (green) in femoral BM sections of WT or β_2-β_3-AR DKO mice of different age. Images were taken from the diaphysis regions; c-kit$^+$CD41$^+$ cells are depicted with white arrows. (E) c-kit$^+$CD41$^+$ cells per mm^2 (n = 4 young mice; n = 3 old mice) in endosteal (e) or non-endosteal (n-e) BM areas. Regions within 1/5 marrow width from the bone surface are defined as endosteal (both sides of the bone), while the remaining 3/5 marrow width in the central part are considered as non-endosteal. (F) Frequency of CD41$^+$ cells in non-endosteal BM Lin$^-$Sca-1$^+$c-kit$^+$ (LSK) HSPCs in endosteal BM from young WT mice (n = 7), young β_2-β_3-AR DKO mice (n = 7), old WT mice (n = 5), and old β_2-β_3-AR DKO mice (n = 4) measured by flow cytometry. (G-J) Peripheral blood counts from young WT mice (n = 8), young β_2-β_3-AR DKO mice (n = 8), old WT mice (n = 6) and old β_2-β_3-AR DKO mice (n = 7). (G) Erythrocytes. (H-J) Frequencies of (H) monocytes, (I) granulocytes and (J) lymphocytes among white blood cells. Data are means ± SEM. * $p < 0.05$; ** $p < 0.01$; *** $p < 0.001$ (One-way Anova followed by Bonferroni pairwise comparisons).

Figure S3, Related to Figure 4

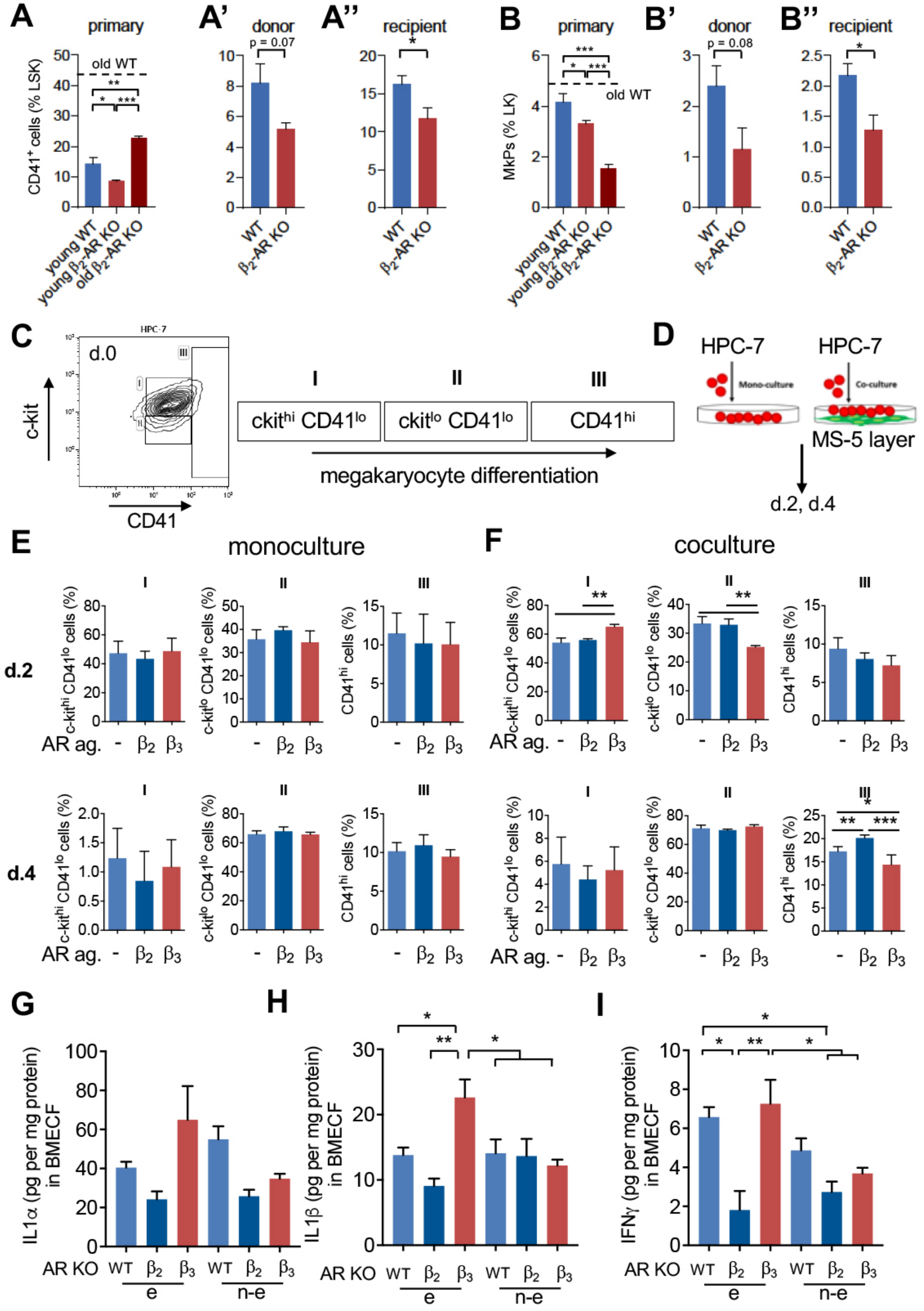

Figure S3, Related to Figure 4. β-ARs have opposite, stage-divergent and stromal-cell-dependent effects on megakaryocyte differentiation. (A-B) Frequency of (A, A', A'') $CD41^+$ myeloid or megakaryocyte progenitors within $lin^-sca\text{-}1^+c\text{-}kit^+$ (LSK) cells or (B, B', B'') $CD150^+CD41^+$ megakaryocyte progenitors (MkPs) within $lin^-c\text{-}kit^+$ (LK) cells in non-endosteal BM cells of the following mice: (A, B) young WT (A, n = 6; B, n = 3), young *Adrb2*$^{-/-}$ (n = 8) or old *Adrb2*$^{-/-}$ (n = 4) mice; (A', B') lethally-irradiated WT recipients of WT (n = 5) or *Adrb2*$^{-/-}$ (n = 4) BM cells; (A'', B'') lethally-irradiated WT (n = 5) or *Adrb2*$^{-/-}$ (n = 4) recipients of WT BM cells. (C) Representative flow cytometry diagram of HPC-7 cells before treatment with cytokines (day 0). Scheme showing the cell populations indicating progressive megakaryocyte commitment: I, $ckit^{hi}CD41^{lo}$ cells; II, $ckit^{lo}CD41^{lo}$ cells; III, $CD41^{hi}$ cells. Megakaryocyte differentiation trajectory flows from populations I → II → III. (D) Scheme showing the experimental design. HPC-7 cells were cultured alone or co-cultured with MS-5 stromal cells in megakaryocyte differentiation medium. (E-F) Quantification of cell populations I, II and III after 2 or 4 days of treatment with vehicle, β_2-AR agonist or β_3-AR agonist in (E) monoculture or (F) coculture with MS-5 stromal layers (n = 4). (G-I) Concentration of (G) IL1α, (H) IL1β and (I) IFNγ in endosteal (e) or non-endosteal (n-e) BM extracellular fluid (BMECF) from adult WT mice (n = 6), *Adrb2*$^{-/-}$ mice (n = 4), or *Adrb3*$^{-/-}$ mice (n = 7). Data are means ± SEM. * $p < 0.05$; ** $p < 0.01$; *** $p < 0.001$. (A, B, E-I) One-way Anova followed by Bonferroni pairwise comparisons. (A', A'', B', B'') Unpaired two-tailed t test.

Figure S4, Related to Figure 5

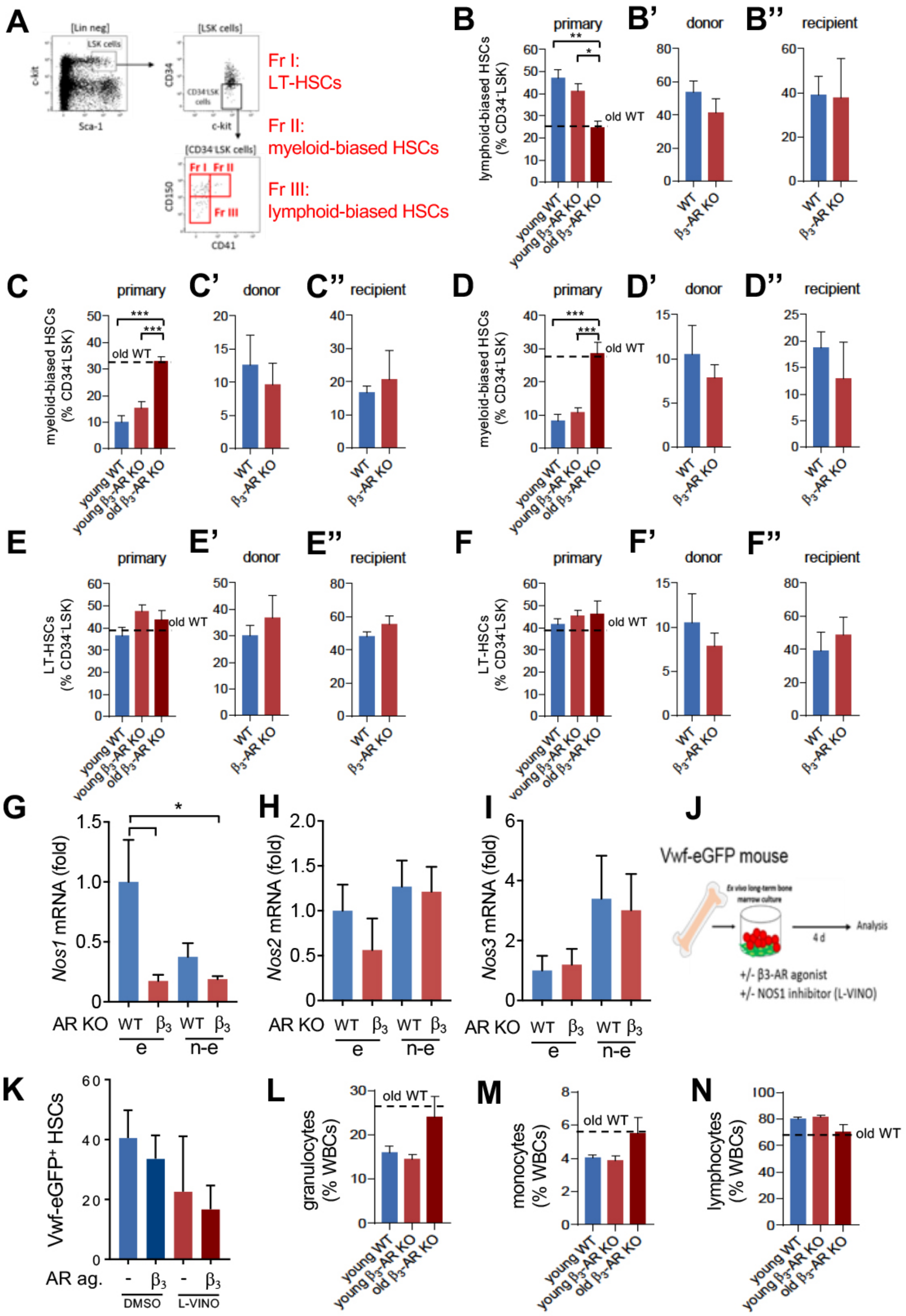

Figure S4, Related to Figure 5. β3-AR regulates HSC lineage bias partially through Nos1-dependent NO production. (A) Representative flow cytometry diagram of HSC populations based on CD41 and CD150 expression. (B) Frequency of $CD150^{lo/-}CD41^-$ lymphoid-biased HSCs in non-endosteal BM $CD34^-$LSK cells. (C-F) Frequency of (C-D) $CD150^+CD41^+$ myeloid-biased HSCs or (E-F) $CD150^+CD41^-$ LT-HSCs in (C, E) endosteal or (D, F) non-endosteal BM $CD34^-$LSK cells from the following mice: (B, C, D, E, F) young WT (n = 7), young *Adrb3*$^{-/-}$ (n = 7) or old *Adrb3*$^{-/-}$ (n = 5) mice; (B', C', D', E', F') lethally-irradiated WT recipients of WT (n = 5) or *Adrb3*$^{-/-}$ (n = 5) BM cells; (B'', C'', D'', E'', F'') lethally-irradiated WT (n = 4) or *Adrb3*$^{-/-}$ (n = 3) recipients of WT BM cells. (G-I) mRNA levels (fold) of (G) *Nos1* (H) *Nos2* and (I) *Nos3* in the endosteal (e) or non-endosteal (n-e) BM of adult WT mice (n = 6) and β_3-AR KO mice (n = 7). (J) Scheme of long-term BM culture from *Vwf-eGFP* mice. (K) Number of $lin^-sca\text{-}1^+c\text{-}kit^+CD34^-CD48^-CD150^+Vwf\text{-}eGFP^+$ platelet-biased HSCs in primary BM culture from *Vwf-eGFP* mice treated with β_3-AR agonist (BRL37344, 10 μM), *Nos1* inhibitor (L-VINO, 100 μM) or vehicle for 4 days (n = 8). (L-N) Frequency of (L) granulocytes, (M) monocytes and (N) lymphocytes in white blood cells (WBCs) in young WT (L, N, n = 10; M, n = 8), young *Adrb3*$^{-/-}$ (n = 4) or old *Adrb3*$^{-/-}$ (n = 6) mice. Data are means ± SEM. * $p < 0.05$; ** $p < 0.01$. (B', B''-F', F'') Unpaired two-tailed *t* test. (B-F, G-N) One-way Anova followed by Bonferroni pairwise comparisons.

Figure S5, Related to Figure 6

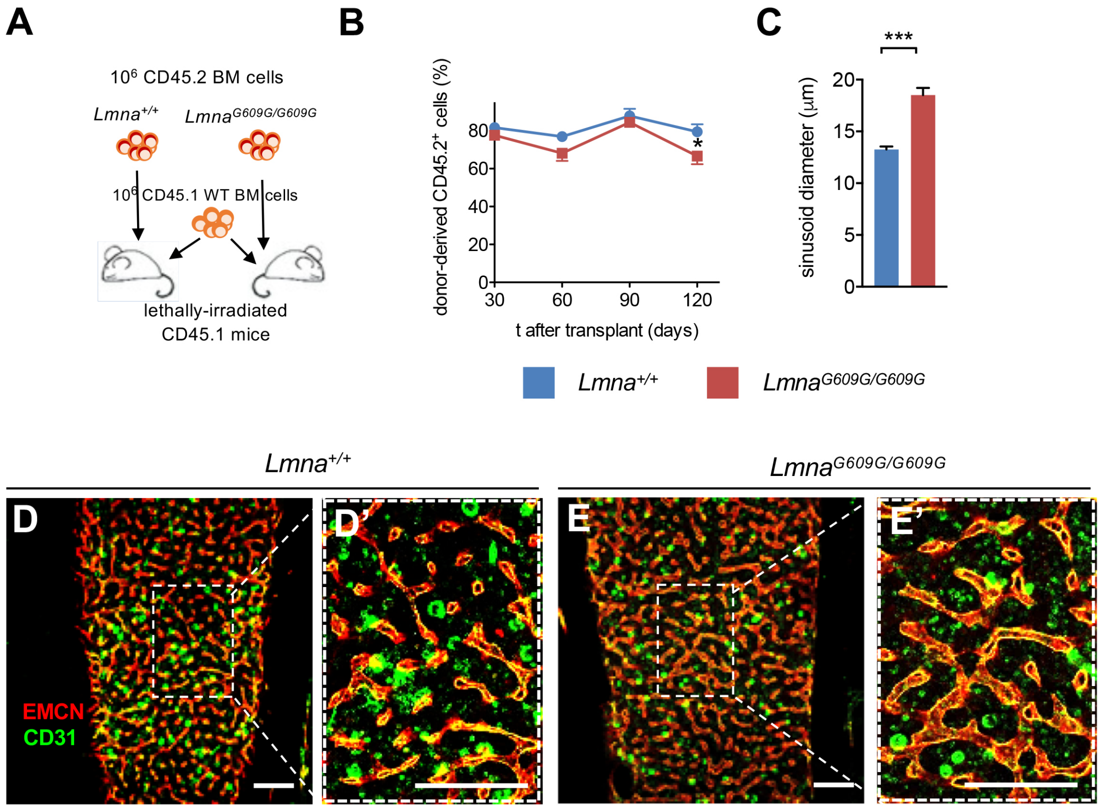

Figure S5, Related to Figure 6. Characterization of hematopoiesis and BM vasculature in HGPS mice. (A) Scheme of competitive BM transplantation. (B) Frequency of CD45.2$^+$ donor-derived hematopoietic cells in peripheral blood of recipient mice 30, 60, 90 and 120 days after lethal irradiation and transplantation with 10^6 BM cells from WT (blue) or *Lmna*$^{G609G/G609G}$ (red) mice, together with 10^6 competitor BM cells from CD45.1 C57BL/6 mice. (C) Quantification of sinusoidal (EMCN$^+$ vasculature) diameter in each group of mice. (D, E) Representative immunofluorescence of CD31 (green) and EMCN (red) in thick femoral BM sections from adult WT mice (*Lmna*$^{+/+}$; n = 7) and *Lmna*$^{G609G/G609G}$ mice (n = 7). Scale bar, 200 µm. (D', E') Insets of D and E. Scale bar, 100 µm. (B-C) Data are means ± SEM. * $p < 0.05$; *** $p < 0.001$ (unpaired two-tailed *t* test).

Figure S6, Related to Figure 6 and Figure 7

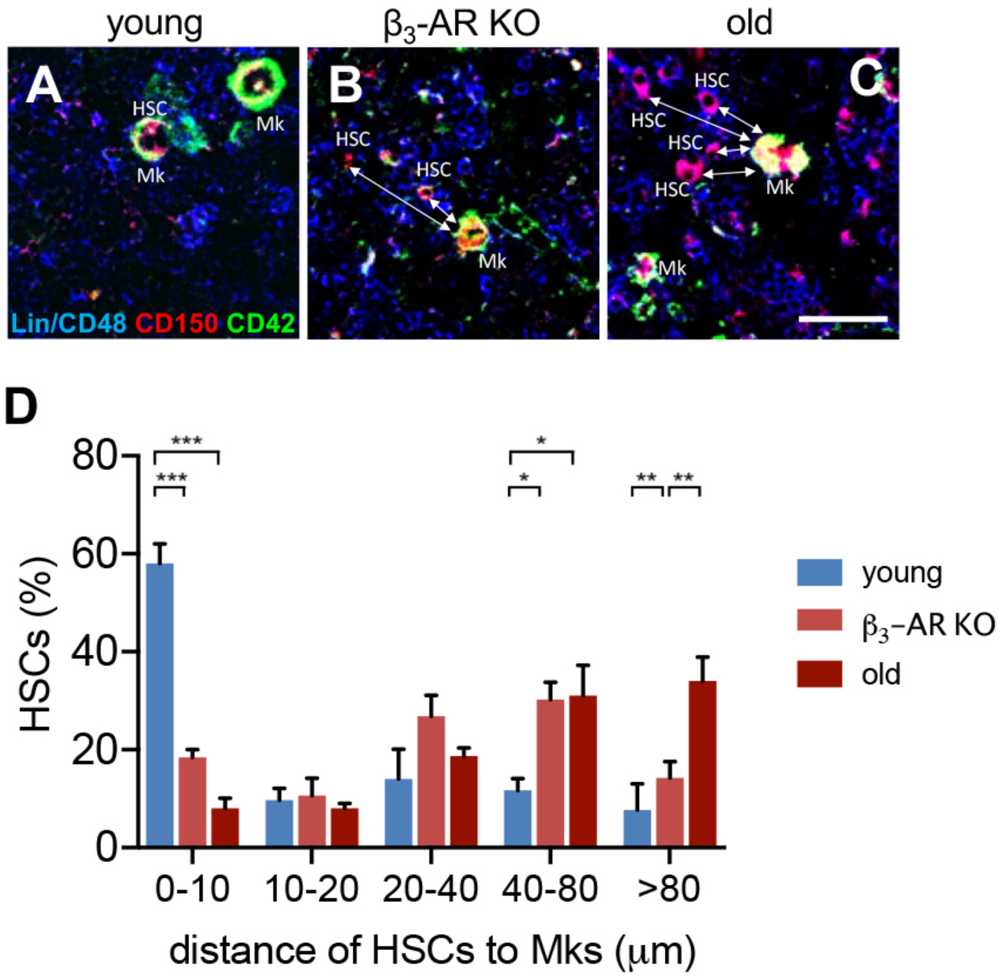

Figure S6, Related to Figure 7. Decreased HSC-megakaryocyte interactions in adult β3-AR KO mice resemble aged WT mice. (A-D) Representative immunofluorescence (A-C) and distribution (D) of BM CD150$^+$ (red) HSCs (negative for mature hematopoietic lineage markers, blue) adjacent (asterisks) or nonadjacent (arrows) to CD42$^+$ (green) megakaryocytes in femoral BM sections of young WT mice (n = 3), adult *Adrb3*$^{-/-}$ mice (n = 5) and old WT mice (n = 3). Scale bar, 50 µm. Data are means ± SEM. * p < 0.05; ** p < 0.01; *** p < 0.001 (Two-way Anova and Bonferroni pairwise comparisons).

Figure S7, Related to Figure 6

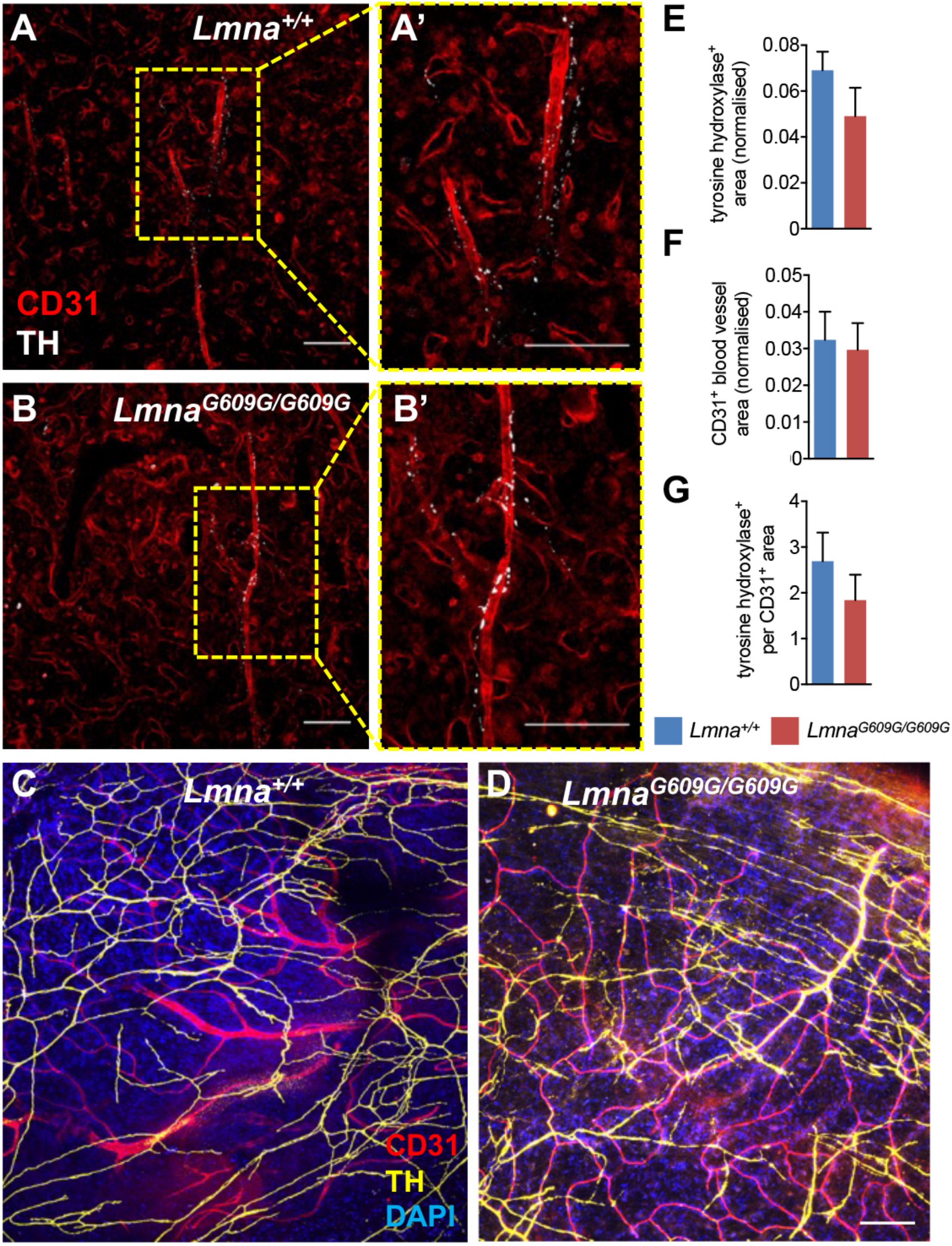

Figure S7, Related to Figure 6 and 7. BM noradrenergic innervation is not reduced in a mouse model of premature aging. (A-D) Whole-mount immunofluorescence staining of tyrosine hydroxylase $(TH)^+$ sympathetic nerve fibers (A, B, white; C, D, yellow) and $CD31^+$ endothelial cells (red) in the femoral BM (A, B) and skull (C, D) of (A, C) adult WT mice (*Lmna*$^{+/+}$; n = 6) and (B, D) *Lmna*$^{G609G/G609G}$ mice (n = 7). A', B', insets of A and B. Scale bar, 100 µm (A, B), 200 µm (C, D). (E) Quantification of the area covered by TH^+ fibers in the skull. (F) Quantification of the area covered by $CD31^+$ endothelial cells in the skull. (G) Ratio of the area of TH^+ fibers to $CD31^+$ endothelial cells in the skull. Data are means ± SEM.
