# Supplementary material for: Remodeling of Bone Marrow Hematopoietic Stem Cell Niches Promotes Myeloid Cell Expansion during Premature or Physiological Aging
Source: Cell Stem Cell. 2019 Sep 5;25(3):407–418.e6. doi: 10.1016/j.stem.2019.06.007 (PMC6739444; doi:10.1016/j.stem.2019.06.007)
Supplement: Document S2. Article plus Supplemental Information [file mmc2.pdf]

Article
Cell Stem Cell

Remodeling of Bone Marrow Hematopoietic Stem Cell Niches Promotes Myeloid Cell Expansion during Premature or Physiological Aging

Graphical Abstract

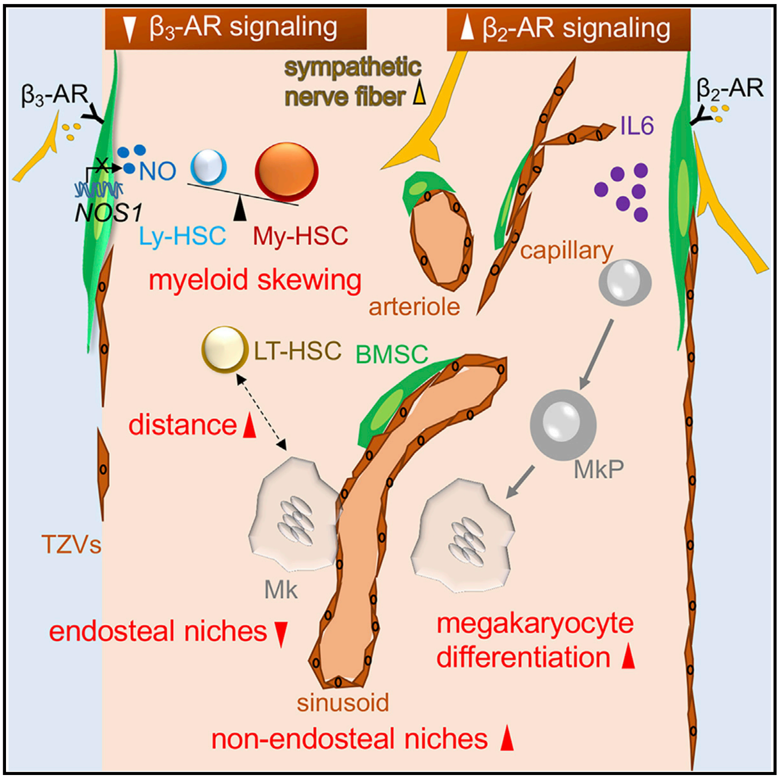

Highlights

- Reduction of endosteal BM and expansion of non-endosteal BM occurs with age
- β2/β3-ARs exhibit opposite and niche-dependent regulation of myelopoiesis
- β2-AR overriding β3-AR promotes myeloid expansion during physiological aging
- Premature HSC aging in HGPS can be improved by targeting the microenvironment

Authors

Ya-Hsuan Ho, Raquel del Toro, José Rivera-Torres, ..., Fawzia Louache, Vicente Andrés, Simón Méndez-Ferrer

Correspondence

sm2116@medschl.cam.ac.uk

In Brief

Recent studies have suggested a microenvironmental contribution to stem-cell aging, but the mechanisms are largely unexplored. Méndez-Ferrer et al. report anatomical remodeling of blood-stem-cell-supporting niches and functional switch of β adrenergic signals, leading to myeloid expansion during aging. Targeting the microenvironment can improve pathological, premature, niche-dependent hematopoietic aging in mice.

Ho et al., 2019, Cell Stem Cell *25*, 407–418
September 5, 2019 © 2019 The Author(s). Published by Elsevier Inc.
https://doi.org/10.1016/j.stem.2019.06.007

CellPress

Cell Stem Cell
Article

CellPress

Remodeling of Bone Marrow Hematopoietic Stem Cell Niches Promotes Myeloid Cell Expansion during Premature or Physiological Aging

Ya-Hsuan Ho,[1,2,13] Raquel del Toro,[3,4,11,13] José Rivera-Torres,[3,4,12,13] Justyna Rak,[1,2] Claudia Korn,[1,2] Andrés García-García,[1,2,3] David Macías,[5] Cristina González-Gómez,[3,4] Alberto del Monte,[3,4] Monika Wittner,[6,7] Amie K. Waller,[1,2] Holly R. Foster,[1,2] Carlos López-Otín,[8,9] Randall S. Johnson,[5] Claus Nerlov,[10] Cedric Ghevaert,[1,2] William Vainchenker,[6] Fawzia Louache,[6,7] Vicente Andrés,[3,4] and Simón Méndez-Ferrer[1,2,3,14,*]

[1]Wellcome Trust-Medical Research Council Cambridge Stem Cell Institute and Department of Haematology, University of Cambridge, Cambridge CB2 0PT, UK
[2]National Health Service Blood and Transplant, Cambridge Biomedical Campus, Cambridge CB2 0PT, UK
[3]Centro Nacional de Investigaciones Cardiovasculares (CNIC), 28029 Madrid, Spain
[4]CIBER de Enfermedades Cardiovasculares (CIBER-CV), Spain
[5]Physiological Laboratory, Department of Physiology, Development and Neuroscience, University of Cambridge, Cambridge CB2 3EG, UK
[6]INSERM (Institut National de la Santé et de la Recherche Médicale), Université Paris-Saclay, UMR1170, Gustave Roussy, 94805 Villejuif, France
[7]Université Paris-Saclay and CNRS GDR 3697 MicroNiT, Villejuif, France
[8]Departamento de Bioquímica y Biología Molecular, Facultad de Medicina, Instituto Universitario de Oncología, Universidad de Oviedo, 33006 Oviedo, Spain
[9]Centro de Investigación Biomédica en Red de Cáncer, CIBERONC, Madrid, Spain
[10]MRC Molecular Haematology Unit, MRC Weatherall Institute of Molecular Medicine, University of Oxford, John Radcliffe Hospital, Headington, Oxford OX3 9DS, UK
[11]Present address: Instituto de Biomedicina de Sevilla-IBiS (Hospital Universitario Virgen del Rocío/CSIC/Universidad de Sevilla), 41013 Seville, Spain
[12]Present address: Universidad Europea de Madrid, Faculty of Biomedical and Health Sciences, Department of Pharmacy, Biotechnology, Nutrition, Optics and Optometry, 28670 Madrid, Spain
[13]These authors contributed equally
[14]Lead Contact
*Correspondence: sm2116@medschl.cam.ac.uk
https://doi.org/10.1016/j.stem.2019.06.007

SUMMARY

Hematopoietic stem cells (HSCs) residing in the bone marrow (BM) accumulate during aging but are functionally impaired. However, the role of HSC-intrinsic and -extrinsic aging mechanisms remains debated. Megakaryocytes promote quiescence of neighboring HSCs. Nonetheless, whether megakaryocyte-HSC interactions change during pathological/natural aging is unclear. Premature aging in Hutchinson-Gilford progeria syndrome recapitulates physiological aging features, but whether these arise from altered stem or niche cells is unknown. Here, we show that the BM microenvironment promotes myelopoiesis in premature/physiological aging. During physiological aging, HSC-supporting niches decrease near bone but expand further from bone. Increased BM noradrenergic innervation promotes β_2-adrenergic-receptor(AR)-interleukin-6-dependent megakaryopoiesis. Reduced β_3-AR-Nos1 activity correlates with decreased endosteal niches and megakaryocyte apposition to sinusoids. However, chronic treatment of progeroid mice with β_3-AR agonist decreases premature myeloid and HSC expansion and restores the proximal association of HSCs to megakaryocytes. Therefore, normal/premature aging of BM niches promotes myeloid expansion and can be improved by targeting the microenvironment.

INTRODUCTION

Hematopoietic aging is characterized by expansion of hematopoietic stem cells (HSCs) with impaired function, such as reduced engraftment, quiescence, self-renewal, unfolded protein response, and lymphoid differentiation potential, leading to myeloid-biased output both in mice (Liang et al., 2005; Mohrin et al., 2015; Rossi et al., 2005; Sudo et al., 2000) and humans (Pang et al., 2011; Rundberg Nilsson et al., 2016). Myeloid malignancies are more frequent in the elderly, but whether changes in the aged HSCs and/or their microenvironment predispose to these malignancies remains unclear. HSC aging was initially considered to result only from intrinsic changes (Dykstra et al., 2011), such as epigenetic deregulation (Chambers et al., 2007), replication stress (Flach et al., 2014), deficient DNA repair (Rossi et al., 2007), transition from canonical to non-canonical Wnt signaling (Florian et al., 2013), and increased autophagy (Ho et al., 2017). However, aging also reduces HSC polarity and capacity to lodge near bone (Köhler et al., 2009), and microenvironmental alterations have been proposed to contribute to hematopoietic aging. For instance, myeloid bias can result from telomere dysfunction (Ju et al., 2007) or inflammatory cytokine production (Ergen et al., 2012) in the aged

This is an open access article under the CC BY license (http://creativecommons.org/licenses/by/4.0/).

Cell Press

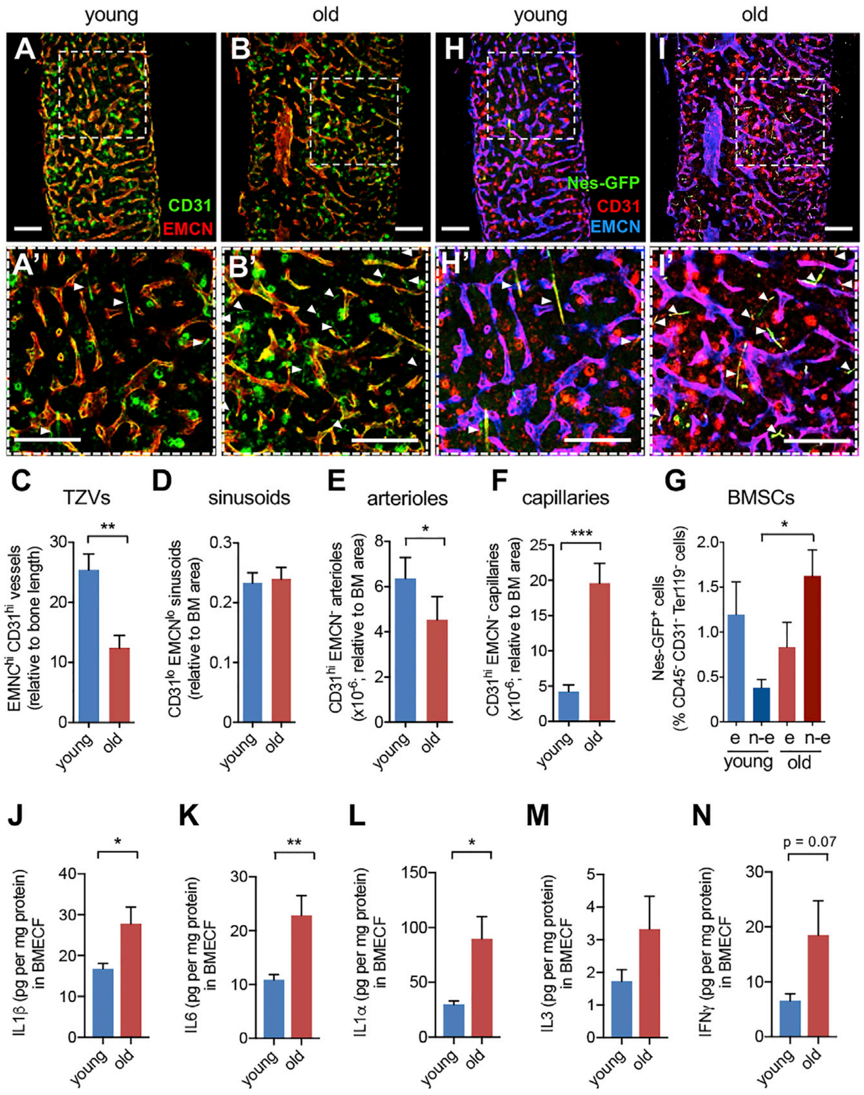

Figure 1. Reduction of Endosteal Niches and Expansion of Non-endosteal Niches during Aging

(A–B′ and H–I′) Representative whole-mount immunofluorescent staining of thick femoral sections for CD31 (A and B, green; H and I, red) and EMCN (A and B, red; H, I, blue) of young (8–30 weeks) and old (70–100 weeks) *Nes-gfp* mice with genetically labeled nestin$^+$ cells (H and I, green). Arrowheads in insets (A′, B′, H′, and I′) depict CD31^{hi}EMCN$^-$ capillaries and their coverage by Nes-GFP$^+$ cells.

(C–G) Quantification of (C) CD31^{hi}EMCN^{hi} transition zone vessels, (D) CD31^{lo}EMCN^{lo} sinusoids, (E) CD31^{hi}EMCN$^-$ arterioles with $\geq$6 μm diameter, and (F) CD31^{hi}EMCN$^-$ capillaries with <6 μm diameter. Scale bar, 200 μm (A, B, H, and I), 100 μm (A′, B′, H′, and I′). (G) Frequency of endosteal and non-endosteal BM Nes-GFP$^+$ cells from young adult (10–20 weeks, n = 11) and old mice (>66 weeks, n = 8).

(J–N) Concentration of (J) IL-1β, (K) IL-6, (L) IL-1α, (M) IL-3, and (N) IFNγ in endosteal BM extracellular fluid (BMECF) from young WT mice (n = 5) and old WT mice (n = 4). Data are means ± SEM. *$p < 0.05$; **$p < 0.01$; ***$p < 0.001$. (C–F and J–N) Unpaired two-tailed t test. (G) One-way ANOVA and Bonferroni pairwise comparisons.

hematopoietic microenvironment, and bone marrow (BM) adrenergic nerve degeneration has been recently proposed to drive HSC aging reversibly (Maryanovich et al., 2018). However, the relative contribution of intrinsic and extrinsic mechanisms to HSC aging remains debated. Myeloid-biased HSCs (Gekas and Graf, 2013) and more prominently platelet-primed HSCs (Sanjuan-Pla et al., 2013; Yamamoto et al., 2013) expand in aged mice (Grover et al., 2016). However, both platelet-primed and unprimed old HSCs exhibit myeloid bias (Grover et al., 2016), possibly suggesting a microenvironmental participation. The standard model of platelet generation (thrombopoiesis) suggests that megakaryocyte precursors migrate from endosteal BM (close to bone) to sinusoids (further from bone) for maturation (Eto and Kunishima, 2016), which can be promoted by cytokines like interleukin (IL)-1α IL-1β, IL-6, IL-3, and interferon-gamma (IFNγ) (Pietras, 2017). Whereas platelet-biased HSCs are located near sinusoids (Pinho et al., 2018), both megakaryocytes and their precursors have been recently found throughout BM, including the sinusoids where megakaryocyte maturation and thrombopoiesis take place (Plo et al., 2017; Stegner et al., 2017). In this study, we have investigated whether different BM microenvironments regulate myeloid differentiation and megakaryopoiesis during aging.

RESULTS

Reduction of Endosteal Niches and Expansion of Non-endosteal Neurovascular Niches during Aging

To characterize changes in vascular beds during aging, we performed whole-mount immunofluorescence staining of thick femoral sections of young and old wild type (WT) mice using CD31 and endomucin (EMCN) to identify sinusoids, arterioles, capillaries, and transition zone vessels (TZVs), which connect arterioles with sinusoids near bone (endosteum). Consistent with previous findings (Kusumbe et al., 2016), endosteal vessels and TZVs were reduced in old mice; however, we also noted that, whereas sinusoidal areas appeared unchanged, arterioles were slightly reduced and capillaries located further from bone increased 4-fold in old mice (Figures 1A–1F). These vascular changes were paralleled by similar alterations of their associated perivascular cells. We used *Nes-gfp* transgenic mice, which carry HSC niche-forming perivascular BM mesenchymal stem or progenitor cells (BMSCs) labeled with GFP (Méndez-Ferrer et al., 2010b). Nes-GFP$^+$ cells augmented 4-fold specifically in non-endosteal BM, mostly associated with the increased capillaries (Figures 1G–1I and S1A–S1D). These changes correlated with increased inflammatory cytokines that drive myeloid cell expansion (Pietras, 2017). The concentration of IL-1α, IL-1β, and IL-6 increased in the BM during aging, whereas IL-3 and IFNγ showed similar trends (Figures 1J–1N and S1M-S1Q).

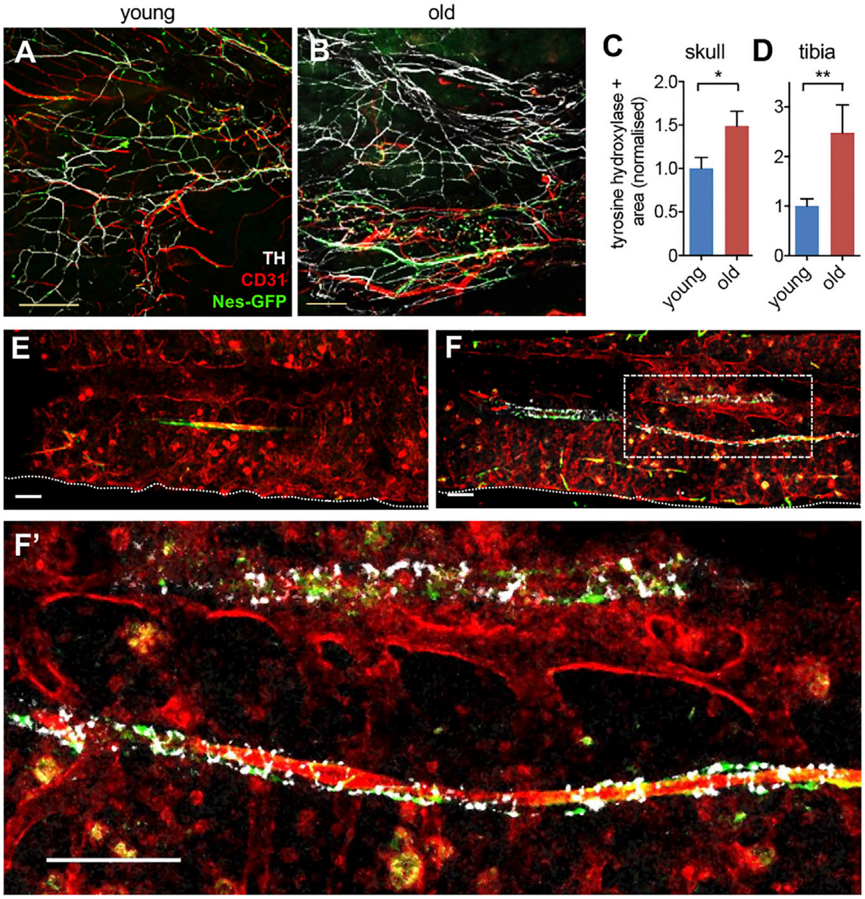

Figure 2. Increased Sympathetic Nerve Fibers during Aging

(A, B, E, and F) Immunofluorescence of tyrosine hydroxylase (TH)$^+$ sympathetic noradrenergic nerve fibers (white), CD31$^+$ endothelial cells (red), and GFP$^+$ cells (green) in the skull (A and B) and tibial (E and F) BM of young (A and E) and old (B and F) *Nes-gfp* mice. Scale bar, 100 μm.

(C and D) Area covered by TH$^+$ fibers in the (C) skull or (D) tibia of young (n = 12) and old (n = 8) *Nes-gfp* mice. Young mice were analyzed between 8–30 weeks of age, and old mice were 66–120 weeks old. Data are means ± SEM. *p < 0.05; **p < 0.01 (unpaired two-tailed t test).

We have previously shown that sympathetic adrenergic signals regulate Nes-GFP$^+$ cell proliferation (Méndez-Ferrer et al., 2010b) and are affected during age-related myeloproliferative neoplasms (Arranz et al., 2014). Additionally, increased sympathetic adrenergic activity has been previously described during aging (Hart and Charkoudian, 2014; Ng et al., 1993; Veith et al., 1986; Ziegler et al., 1976), chronic stress, and depression (Yirmiya et al., 2006), and might increase osteoporosis and fracture risk by restraining bone formation (Elefteriou et al., 2005; Takeda et al., 2002). However, the opposite (decreased BM adrenergic innervation) has been recently suggested as causative of HSC aging (Maryanovich et al., 2018). To clarify this, whole-mount preparations of skulls and thick tibial sections of *Nes-gfp* mice were immunostained for tyrosine hydroxylase (TH), to visualize sympathetic noradrenergic fibers and nestin$^+$ cells in large 3D volumes. This study did not confirm reduced TH$^+$ fibers in the aged BM (Maryanovich et al., 2018) but found these fibers increased by 50% in the skull of old mice (Figures 2A–2C) and augmented 2.5-fold in the aged tibial BM, compared with the young samples (Figures 2D–2F). In both cases, nestin$^+$ cells were found in proximity of noradrenergic fibers (Figures S1E–S1L). Together, these results suggest contraction of endosteal (bone-associated) HSC niches and expansion of non-endosteal neurovascular HSC niches during aging.

β-Adrenergic Signals Promote Megakaryopoiesis during Aging

To study the possible contribution of increased adrenergic innervation to aged hematopoiesis, we analyzed mice lacking β_2-AR and β_3-AR (*Adrb2*$^{-/-}$*Adrb3*$^{-/-}$), the main β-ARs that cooperate in HSC niche regulation (Méndez-Ferrer et al., 2010a). Importantly, myeloid and megakaryocyte progenitors expanded less in the endosteal BM of aged *Adrb2*$^{-/-}$*Adrb3*$^{-/-}$ mice (Figures 3A, 3B, and S2A–S2F). Consequently, megakaryocytes did not increase in *Adrb2*$^{-/-}$*Adrb3*$^{-/-}$ mice during aging (Figures 3C–3G), suggesting a functional role for increased noradrenergic signaling in aged hematopoiesis. Megakaryocytes were less frequently found in apposition to BM sinusoids and could form less protrusions (required for proplatelet formation) in aged *Adrb2*$^{-/-}$*Adrb3*$^{-/-}$ mice (Figures 3H–3M). Accordingly, aged *Adrb2*$^{-/-}$*Adrb3*$^{-/-}$ mice did not show the increased circulating platelets typically observed in aged WT mice (Figure 3N), whereas other circulating blood cell types remained unaffected (Figures S2G–S2J). These results suggest that increased β-adrenergic signaling in the BM promotes myeloid differentiation into platelets during aging.

β_2-AR in the Microenvironment Promotes Megakaryocyte Differentiation

We analyzed single *Adrb2*$^{-/-}$ mice and *Adrb3*$^{-/-}$ mice to understand the role of each β-AR in myelopoietic regulation. Resembling aged *Adrb2*$^{-/-}$*Adrb3*$^{-/-}$ mice (Figure 3N), aged *Adrb2*$^{-/-}$ mice did not exhibit increased circulating platelets (Figure 4A), suggesting that increased β_2-adrenergic activity promotes thrombopoiesis during aging. Already at adulthood, *Adrb2*$^{-/-}$ mice showed reduced frequency of myeloid and megakaryocyte progenitors, and this reduction persisted during aging (Figures 4B, 4C, S3A, and S3B). We generated BM chimeras using *Adrb2*$^{-/-}$ mice or WT mice as donors/recipients to distinguish hematopoietic cell-autonomous from microenvironmental regulation. WT mice carrying β_2-AR-deficient hematopoietic cells showed normal frequencies of myeloid and megakaryocyte progenitors (Figures 4B′–4C′

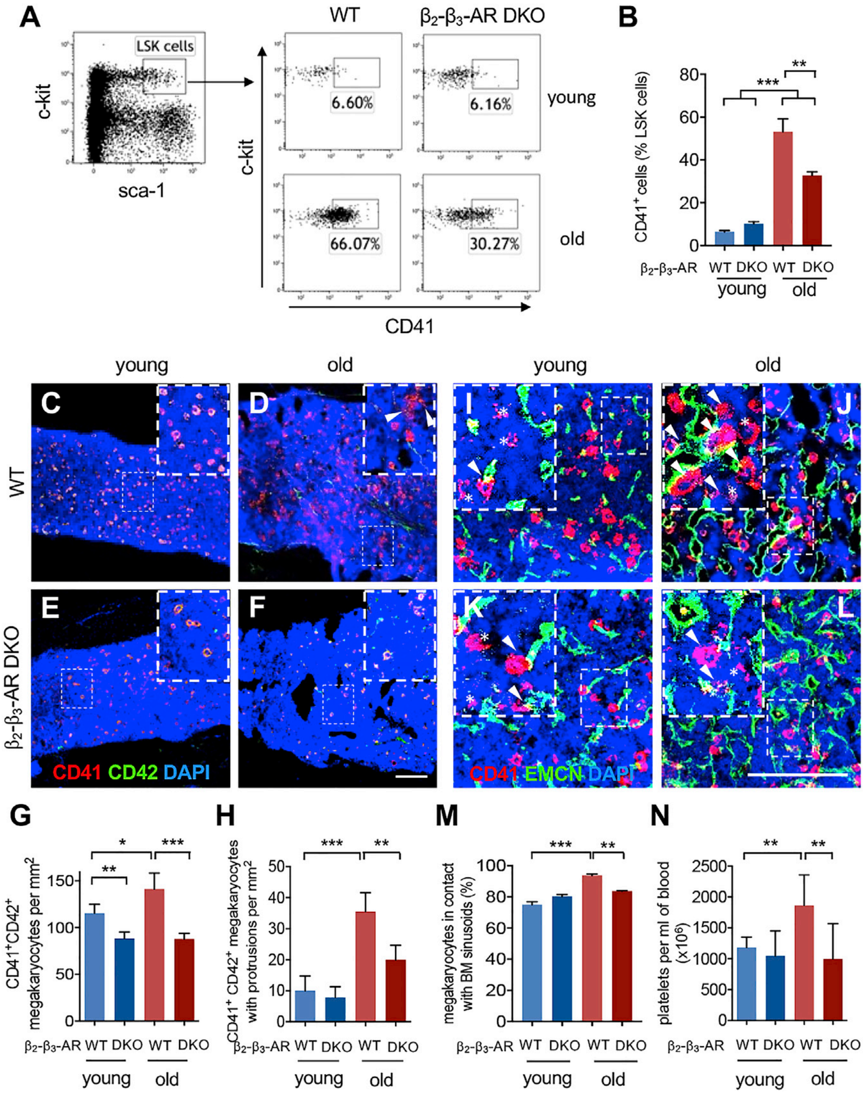

Figure 3. β-Adrenergic Signals Promote Megakaryopoiesis during Aging

(A and B) Representative flow chart (A) and quantification (B) of the frequency of $CD41^+$ myeloid or megakaryocyte progenitors within $lin^-sca\text{-}1^+c\text{-}kit^+$ (LSK) cells in endosteal BM from young WT mice (n = 7), young *Adrb2*$^{-/-}$*Adrb3*$^{-/-}$ (double knockout [DKO]) mice (n = 7), old WT mice (n = 5), and old *Adrb2*$^{-/-}$*Adrb3*$^{-/-}$ mice (n = 4).

(C–F) Representative immunofluorescence staining for CD41 (red) and CD42 (green) in femoral BM sections of young (C) or old (D) WT mice and young (E) or old (F) *Adrb2*$^{-/-}$*Adrb3*$^{-/-}$ mice. Arrowheads depict megakaryocytes with protrusions ($CD41^+$ $CD42^+$ cells with cell body extensions). Scale bar, 250 μm.

(G and H) Number of $CD41^+CD42^+$ megakaryocytes (G) forming protrusions (H) per BM area (n = 4 young WT; n = 5 young DKO; n = 3 old mice).

(I–L) Representative immunofluorescence staining for CD41 (red) and EMCN (green) in femoral BM sections of young (I) or old (J) WT mice and young (K) or old (L) *Adrb2*$^{-/-}$*Adrb3*$^{-/-}$ mice, depicting $CD41^+$ megakaryocytes adjacent (arrowheads) or nonadjacent (asterisks) to $EMCN^+$ vasculature. Scale bar, 100 μm.

(M) Frequency of $CD41^+$ cells in contact with $EMCN^+$ vasculature (n = 4 young WT; n = 4 young DKO; n = 4 old WT; n = 3 old DKO).

(N) Circulating platelets in young (n = 11) and old (n = 6) WT mice, compared with young (n = 9) and old (n = 5) *Adrb2*$^{-/-}$*Adrb3*$^{-/-}$ mice. Young mice were analyzed between 8–30 weeks of age, and old mice were 66–120 weeks old. Data are means ± SEM. *$p < 0.05$; **$p < 0.01$; ***$p < 0.001$ (one-way ANOVA followed by Bonferroni pairwise comparisons).

and S3A′–S3B′). In contrast, *Adrb2*$^{-/-}$ recipients of WT BM cells showed similar reductions as found in primary mice (Figures 4B″–4C″ and S3A″–S3B″). These results suggest that β_2-AR signals promote megakaryocyte differentiation through the microenvironment. To obtain mechanistic insight, we treated with selective β_2-AR or β_3-AR agonists the HSPC-like HPC-7 cell line (Pinto do O et al., 1998) cultured alone or co-cultured with MS-5 stromal cells, which resemble $nestin^+$ BMSCs (Méndez-Ferrer et al., 2008, 2010b; Figures S3C and S3D). Whereas the selective β-AR agonists did not affect megakaryocyte differentiation of HPC-7 cells cultured alone (Figure S3E), β_2-AR and β_3-AR agonists had opposite stage-specific effects on megakaryocytic differentiation from HPC-7 cells co-cultured with MS-5 cells. Whereas β_3-AR agonist increased the frequency of undifferentiated $c\text{-}kit^{hi}$ $CD41^{lo}$ cells and decreased the fraction of $c\text{-}kit^{lo}CD41^{lo}$ megakaryocyte progenitors, β_2-AR agonist did not affect early differentiation, but instead increased the frequency of $CD41^{hi}$ megakaryocytic cells at a later differentiation stage (Figure S3F).

To test the potential significance of this regulation in the human setting, we treated human umbilical cord blood-derived $hCD34^+$ HSPCs co-cultured with MS-5 cells with a selective β_2-AR agonist (Figure 4D). Treatment with β_2-AR agonist increased the frequency of human $CD61^+$ megakaryocytic cells (Figures 4E–4G). These results suggest that β_2-AR stimulation of stromal cells promotes megakaryocyte differentiation.

β_2-AR Indirectly Promotes Megakaryocyte Differentiation through IL-6

To investigate the underlying mechanism, we measured several cytokines which regulate megakaryopoiesis. Among those, IL-6 concentration increased in WT BM during aging (Figure 1H) and was low in endosteal *Adrb2*$^{-/-}$ BM (Figures 4H and S3G–S3I). Moreover, β_2-AR agonist increased *Il6* mRNA 2-fold in MS-5 stromal cells; this effect was abrogated after blocking protein kinase A (Figure 4I) downstream of β_2-AR signaling (Rosenbaum et al., 2009). Adult *Adrb2*$^{-/-}$ mice and *Il6*$^{-/-}$ mice showed similarly reduced BM megakaryocytes (Figures 4J–4M), confirming the role of β_2-AR and IL6 in megakaryopoiesis. To directly test the role of IL-6 downstream of β_2-AR, we established primary BM cultures from *Il6*$^{-/-}$ or control mice and treated them with

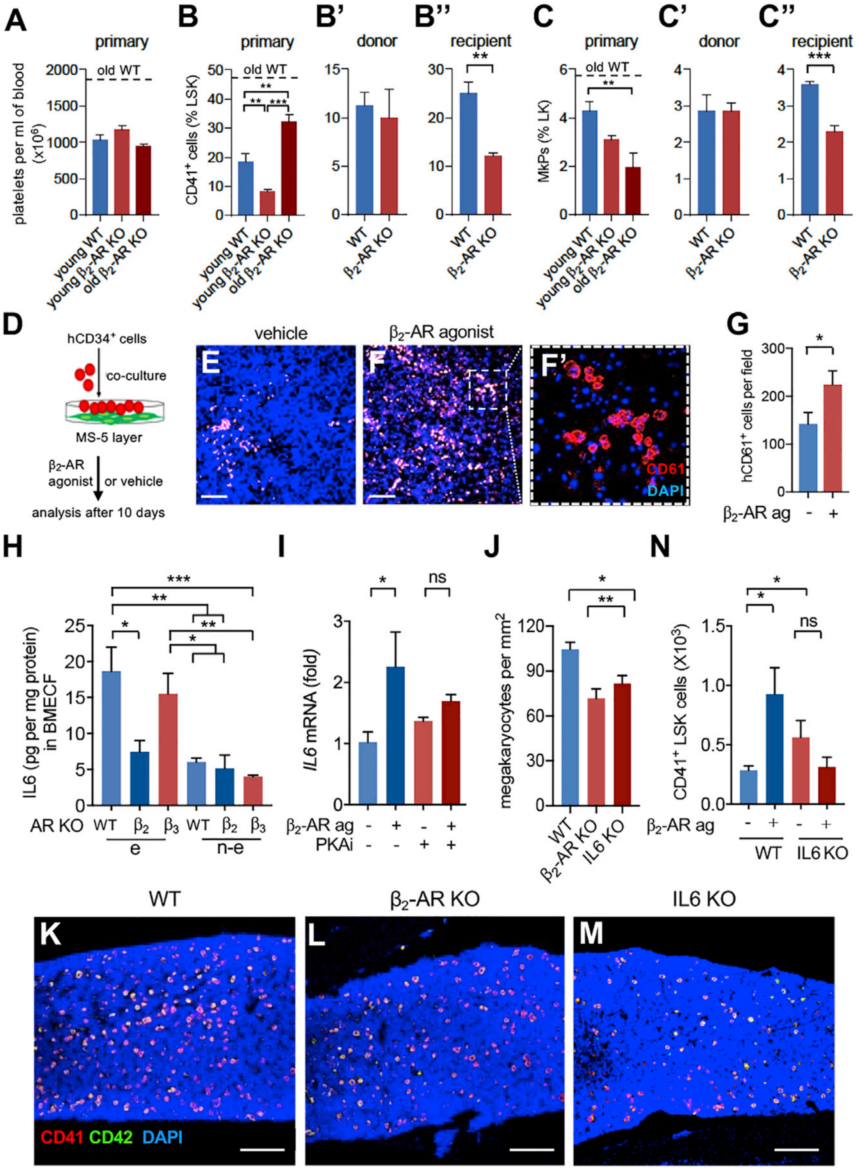

Figure 4. β_2-AR Signaling in the Microenvironment Promotes Megakaryocyte Differentiation through IL-6

(A) Circulating platelets in young WT (n = 11), young *Adrb2*$^{-/-}$ (n = 3), or old *Adrb2*$^{-/-}$ (n = 3) mice.

(B–C″) Frequency of (B, B′, and B″) CD41^{+} myeloid or megakaryocyte progenitors within lin^{-}sca-1^{+}c-kit^{+} (LSK) cells or (C, C′, and C″) CD150^{+}CD41^{+} megakaryocyte progenitors (MkPs) within lin^{-}c-kit^{+} (LK) cells in endosteal BM cells of the following mice: (B and C) young WT (B, n = 6; C, n = 3), young *Adrb2*$^{-/-}$ (B, n = 6; C, n = 8) or old *Adrb2*$^{-/-}$ (n = 4) mice; (B′ and C′) lethally irradiated WT recipients of WT (n = 5) or *Adrb2*$^{-/-}$ (n = 4) BM cells; (B″ and C″) lethally irradiated WT (n = 5) or *Adrb2*$^{-/-}$ (n = 4) recipients of WT BM cells.

(D) Scheme of human umbilical-cord-blood-derived CD34^{+} HSPCs cocultured with MS-5 stromal cells.

(E–G) Representative immunofluorescence (E and F) and number (G) of CD61^{+} (red) human megakaryocytes in cocultures treated with (E) vehicle or (F) β_2-AR agonist (clenbuterol, 10 μM) for 10 days (n = 3). Scale bar, 250 μm. (F′) Inset of (F).

(H) IL6 concentration in endosteal (e) or non-endosteal (n-e) BM supernatant from adult WT (n = 6), *Adrb2*$^{-/-}$ (n = 4), or *Adrb3*$^{-/-}$ (n = 7) mice.

(I) *Il6* mRNA expression (fold change) in MS-5 stromal cells treated with β_2-AR agonist (clenbuterol, 10 μM), PKA inhibitor (H-89, 5 μM), or vehicle for 2 days (n = 3).

(J–M) Quantification (J) and representative immunofluorescence (K–M) of CD41^{+} (red) CD42^{+} (green) megakaryocytes (yellow) in adult WT (n = 5), *Adrb2*$^{-/-}$ (n = 3), or *Il6*$^{-/-}$ (n = 5) mice. Scale bar, 250 μm.

(N) Myeloid or megakaryocyte progenitors (CD41^{+} LSK cells) in primary BM culture from WT or *Il6*$^{-/-}$ mice treated with β_2-AR agonist (clenbuterol, 10 μM) or vehicle for 4 days (n = 4). Data are means ± SEM. *p < 0.05; **p < 0.01; ***p < 0.001. (B′, B″, C′, C″, and G) Unpaired two-tailed t test. (A, B, C, H, I, J, and N) one-way ANOVA and Bonferroni pairwise comparisons.

β_2-AR agonist or vehicle. β_2-AR agonist tripled myeloid progenitors in WT samples, but not in *Il6*$^{-/-}$ samples (Figure 4N). Altogether, these results suggest that β_2-AR on stromal cells promotes megakaryocyte differentiation through IL-6.

β_3-AR-Deficient Mice Exhibit Altered HSC Lineage Bias

Our co-culture experiments suggested that, contrasting β_2-AR, β_3-AR might inhibit megakaryocytic differentiation (Figures S3C–S3F). Additionally, β_3-AR stimulation has been shown to promote HSC lymphoid skewing (Maryanovich et al., 2018). Therefore, we measured the frequency of immunophenotypically defined lymphoid-biased HSCs (Figure S4A; Yamamoto et al., 2013). Lymphoid-biased HSCs were reduced by one-third in adult endosteal *Adrb3*$^{-/-}$ BM (Figures 5A and S4B). This phenotype was not observed in WT mice carrying β_3-AR-deficient hematopoietic cells (Figures 5A′ and S4B′) but was reproduced in chimeric mice lacking β_3-AR in the microenvironment (Figures 5A″ and S4B″), implying niche-mediated regulation. Opposite trends were observed for long-term HSCs (LT-HSCs) and myeloid-biased HSCs, with their frequencies tending to increase in endosteal *Adrb3*$^{-/-}$ BM (Figures S4C–S4F). These results suggest accelerated lymphoid deficiency in the endosteal microenvironment lacking β_3-AR.

We investigated possible mechanisms that might explain the intriguingly opposite effects of β_3-AR and β_2-AR signaling on lympho-myeloid lineage skewing. Whereas both β-ARs can activate G proteins (Rosenbaum et al., 2009), their differential cardiovascular effects have been attributed to β_3-AR-dependent nitric oxide (NO) generation (Gauthier et al., 1998). Therefore, we measured nitrate concentration in the BM extracellular fluid and found it doubled in endosteal (compared with non-endosteal) WT BM and halved in *Adrb3*$^{-/-}$ BM (Figure 5B). Among NO synthases, *Nos1* (but not *Nos2* or *Nos3*) showed higher mRNA expression in endosteal WT BM and was downregulated in

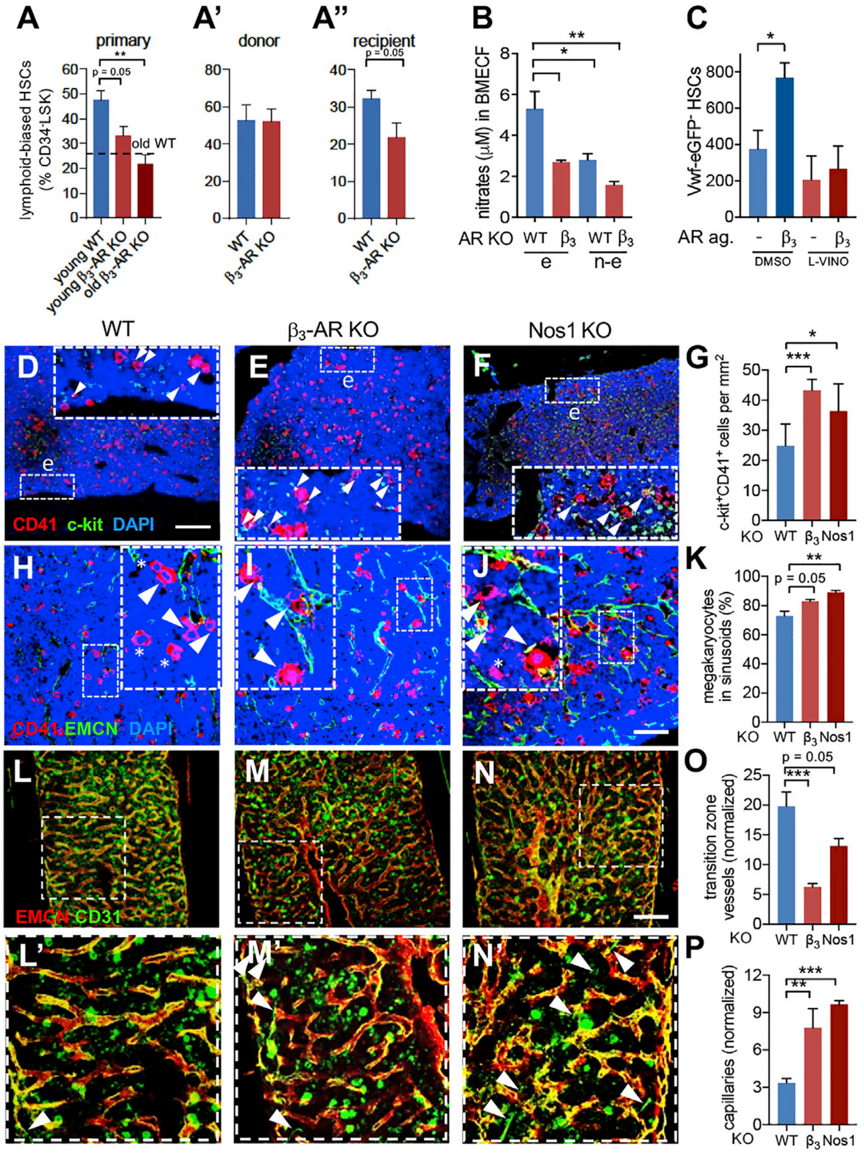

Figure 5. Lack of β_3-AR and Nitric Oxide Synthase 1 (Nos1) Signaling in the Microenvironment Accelerates Aging

(A, A′, and A″) Frequency of $CD150^{lo/-}CD41^{-}$ lymphoid-biased HSCs in endosteal BM $CD34^{-}$LSK cells from the following mice: (A) young WT (n = 7), young *Adrb3*$^{-/-}$ (n = 7), or old *Adrb3*$^{-/-}$ (n = 5) mice; (A′) lethally irradiated WT recipients of WT (n = 5) or *Adrb3*$^{-/-}$ (n = 5) BM cells; and (A″) lethally irradiated WT (n = 4) or *Adrb3*$^{-/-}$ (n = 3) recipients of WT BM cells.

(B) Nitrate concentration in BM extracellular fluid (BMECF) of young WT (n = 6) or *Adrb3*$^{-/-}$ (n = 7) mice.

(C) Number of lin^{-}sca-1^{+}c-kit^{+}CD34^{-}CD48^{-}CD150^{+}Vwf-eGFP^{-} lymphoid-biased HSCs in primary BM culture from *Vwf-eGFP* mice treated with β_3-AR agonist (BRL37344, 10 μM), *Nos1* inhibitor (L-VINO, 100 μM), or vehicle for 4 days (n = 8).

(D–G) Representative immunofluorescence (D–F) and quantification (G) of c-kit^{+} (green) CD41^{+} (red) myeloid or megakaryocyte progenitors (arrowheads) in endosteal BM of WT (n = 10), *Adrb3*$^{-/-}$ (n = 8), or *Nos1*$^{-/-}$ (n = 4) mice. Endosteal BM is considered as regions within one-fifth marrow width from the bone surface. Scale bar, 300 μm.

(H–K) Representative immunofluorescence (H-J) and quantification (K) of CD41^{+} (red) megakaryocytes adjacent (arrowheads) or nonadjacent (asterisks) to EMCN^{+} (green) BM vasculature of WT (n = 9), *Adrb3*$^{-/-}$ (n = 6), or *Nos1*$^{-/-}$ (n = 4) mice. Scale bar, 250 μm.

(L–N′) Representative whole-mount immunofluorescence of CD31 (green) and EMCN (red) in WT (n = 7), *Adrb3*$^{-/-}$ (n = 5), or *Nos1*$^{-/-}$ (n = 4) BM. Arrowheads in insets (L′), (M′), and (N′) depict CD31^{hi}EMCN^{-} capillaries.

(O and P) Quantification of (O) CD31^{hi}EMCN^{hi} transition zone vessels and (P) CD31^{hi}EMCN^{-} capillaries with <6 μm diameter. Scale bar, 250 μm. Data are means ± SEM. *p < 0.05; **p < 0.01; ***p < 0.001. (A′ and A″) Unpaired two-tailed t test. (A–C, G, K, O, and P) One-way ANOVA and Bonferroni pairwise comparisons.

Adrb3$^{-/-}$ BM (Figures S4G–S4I). To further study the role of β_3-AR and Nos1 on HSC lineage bias, we established primary BM cultures from transgenic mice expressing GFP under the regulatory elements of Von Willebrand factor (*Vwf-eGFP*), which discriminate myeloid/platelet-biased (Vwf-eGFP^{+}) HSCs from lymphoid-biased (Vwf-eGFP^{-}) HSCs (Sanjuan-Pla et al., 2013). Treatment with β_3-AR agonist did not affect platelet-biased HSCs (Figures S4J and S4K) but doubled lymphoid-biased HSCs in a Nos1-dependent manner (Figure 5C). Moreover, *Adrb3*$^{-/-}$ mice and *Nos1*$^{-/-}$ mice showed similar signs of premature hematopoietic aging; myeloid or megakaryocyte progenitors (Figures 5D–5G) and megakaryocyte apposition to blood vessels (Figures 5H–5K) similarly increased in adult *Adrb3*$^{-/-}$ mice and *Nos1*$^{-/-}$ mice. Consistently, transition zone vessels decreased (Figures 5L–5O) whereas capillaries expanded (Figure 5P) in both KO models. Despite the partial reduction of lymphoid-biased HSCs in endosteal *Adrb3*$^{-/-}$ BM, the frequencies of circulating lymphocytes or myeloid cells appeared unchanged in 5-month-old *Adrb3*$^{-/-}$ mice (Figures S4L–S4N), contrasting previous findings (Maryanovich et al., 2018). Whereas adult *Adrb3*$^{-/-}$ mice did not yet show in our analysis premature hematopoietic aging in peripheral blood, adult *Nos1*$^{-/-}$ mice showed reduced lymphocytes and increased neutrophils and platelets in circulation (Figures 6A and 6B), suggesting that other pathways (besides β_3-AR) contribute to Nos1-dependent regulation of hematopoiesis. Altogether, these results suggest that microenvironmental β_3-AR contributes to balance HSC lineage-bias toward lymphoid production, which is at least partially dependent on Nos1-dependent NO production.

Premature Hematopoietic Aging in HGPS Is Not HSC-Autonomous

We next interrogated whether the BM microenvironment might promote pathological (and not only physiological) hematopoietic

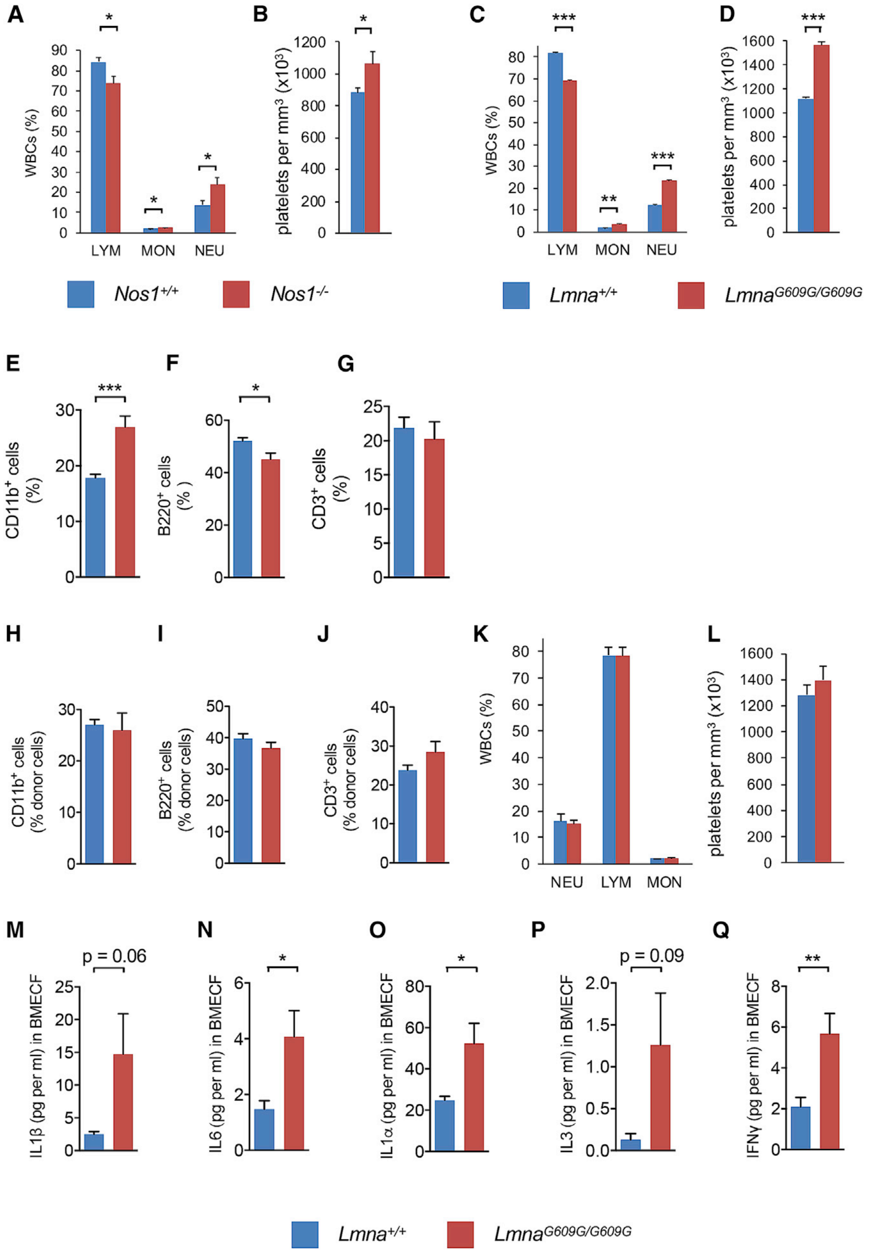

Figure 6. Premature Hematopoietic Aging in HGPS Is Not HSC-Autonomous

(A–D) Peripheral blood counts in adult (A and B) WT (*Nos1*$^{+/+}$; n = 15) or *Nos1*$^{-/-}$ (n = 16), and (C and D) WT (*Lmna*$^{+/+}$; n = 22) or *Lmna*$^{G609G/G609G}$ (n = 14) mice. (A, C, and K) Frequency of white blood cells (WBC); LYM, lymphocytes; MON, monocytes; NEU, neutrophils. (B, D, and L) Concentration of platelets.

(E–G) Frequency of (E) CD11b$^+$ myeloid cells, (F) B220$^+$ B cells, and (G) CD3$^+$ T cells in circulating leukocytes.

(H–J) Frequency of (H) CD11b$^+$ myeloid cells, (I) B220$^+$ B cells, and (J) CD3$^+$ T cells among donor-derived leukocytes 120 days after transplantation into WT mice (n = 11).

(K and L) Peripheral white blood cell (K) and platelet (L) counts in adult CD45.1 C57BL/6J mice 4 months after lethal irradiation and transplantation with CD45.2 BM cells from WT (n = 8) or *Lmna*$^{G609G/G609G}$ (n = 6) mice.

(M–Q) Concentration of (M) IL-1β, (N) IL-6, (O) IL-1α, (P) IL-3, and (Q) IFNγ in the BM extracellular fluid (BMECF) of adult WT (*Lmna*$^{+/+}$; n = 9) and *Lmna*$^{G609G/G609G}$ (n = 9) male mice. Data are means ± SEM. *p < 0.05; **p < 0.01; ***p < 0.001 (unpaired two-tailed t test).

aging. Premature aging in Hutchinson-Gilford progeria syndrome (HGPS) is caused by the accumulation of a truncated prelamin A protein named “progerin,” which is produced via aberrant splicing resulting from a *de novo* synonymous c.1824C > T (p.G608G) point mutation in the *LMNA* gene (encoding lamin A and C) (De Sandre-Giovannoli et al., 2003; Eriksson et al., 2003). This gene has been recently associated with changes in epigenetic and chromatin architecture in aged HSCs (Grigoryan et al., 2018). Progerin also accumulates during normal aging and HGPS displays most aging hallmarks, suggesting that HGPS can inform physiological aging (López-Otín et al., 2013). *Lmna*^{G609G} knockin mice exhibit key hallmarks of the human disease, including accelerated aging, shortened lifespan, and bone and cardiovascular defects (Hamczyk et al., 2018; Osorio et al., 2011; Villa-Bellosta et al., 2013). Certain hallmarks of hematopoietic aging in mice, such as increased circulating platelets, have been observed in HGPS (Merideth et al., 2008). However, it remains unknown whether premature aging affects the overall hematopoietic system in HGPS and whether this is a consequence of the *LMNA* mutation in HSCs, other hematopoietic cells and/or their microenvironment.

We measured peripheral blood counts in progeroid mice and control mice. Remarkably, adult progeroid mice resembled adult *Nos1*$^{-/-}$ mice (Figures 6A and 6B) in their premature myeloid skewing evidenced by decreased lymphocytes and increased neutrophils, monocytes, and platelets (Figures 6C and 6D), to a similar extent as normally aged mice (Dykstra et al., 2011; Rossi et al., 2005; Sudo et al., 2000). Flow cytometry analysis confirmed increased myeloid cells and decreased lymphoid cells in the peripheral blood of progeroid mice (Figures 6E–6G).

To test HSC function, lethally irradiated CD45.1 recipients were transplanted with CD45.2 BM cells from *Lmna*$^{G609G/G609G}$ mice or WT mice, together with competitor BM cells from congenic CD45.1 mice (Figure S5A). Long-term engraftment was lower for *Lmna*$^{G609G/G609G}$ (compared with WT) hematopoietic cells (Figure S5B), as previously observed for normally aged HSCs (Liang et al., 2005), albeit to a lower extent. However, myeloid skewing was not observed in blood cells derived from

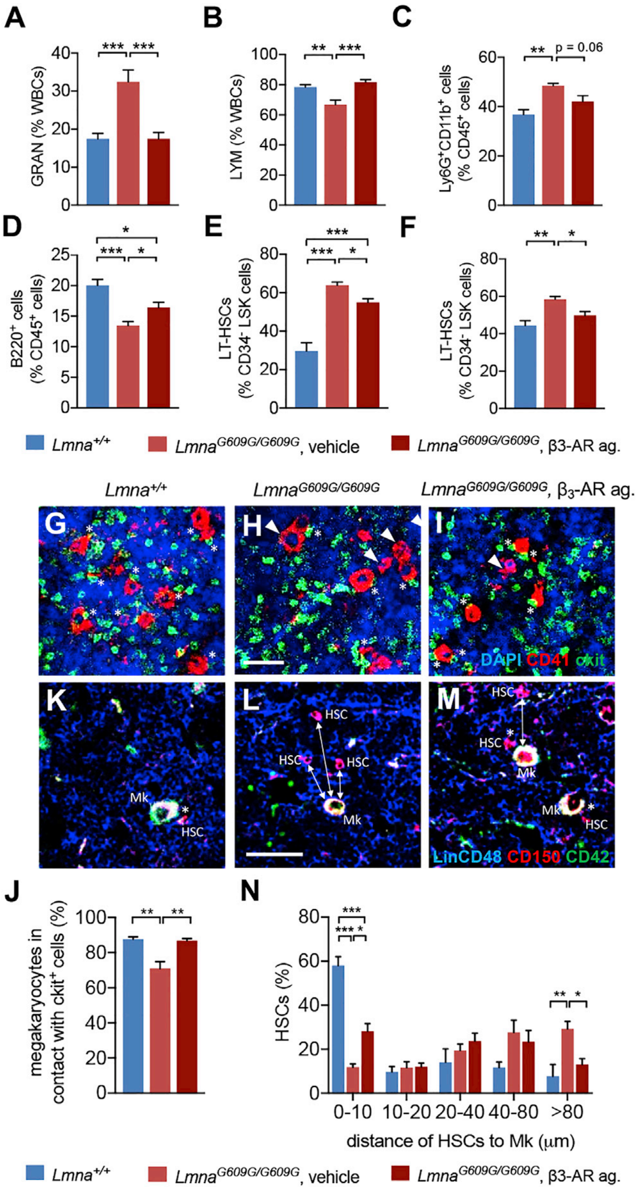

Figure 7. β_3-AR Agonist Improves Lineage Skewing, HSC Number, and Localization near Megakaryocytes in HGPS

(A and B) Frequency of (A) granulocytes and (B) lymphocytes in white blood cells (WBCs) of WT mice (n = 8) or *Lmna*$^{G609G/G609G}$ mice (n = 6) treated with β_3-AR agonist (BRL37344, 2 mg/kg/day, intraperitoneally [i.p.]) or vehicle for 8 weeks.

(C–F) Frequency of BM (C) Ly6G$^+$CD11b$^+$ neutrophils, (D) B220$^+$ B cells, endosteal (E), and non-endosteal (F) LT-HSCs in these mice (n = 6).

(G–J) Representative immunofluorescence (G–I) and quantification (J) of CD41$^+$ megakaryocytes adjacent (asterisks) or nonadjacent (arrowheads) to c-kit$^+$ (green) HSPCs in BM of WT mice (n = 3) and *Lmna*$^{G609G/G609G}$ mice (n = 5) treated with β_3-AR agonist or vehicle. Scale bar, 50 μm.

(K–N) Representative immunofluorescence (K–M) and distribution (N) of BM CD150$^+$ (red) HSCs (negative for mature hematopoietic lineage markers, blue) adjacent (asterisks), or nonadjacent (arrows) to CD42$^+$ (green) megakaryocytes. Scale bar, 50 μm. Data are means ± SEM. *$p < 0.05$; **$p < 0.01$; ***$p < 0.001$. (A–F and J) One-way ANOVA and Bonferroni pairwise comparisons. (N) Two-way ANOVA and Bonferroni pairwise comparisons.

Lmna$^{G609G/G609G}$ HSCs (Figures 6H–6J), suggesting that myeloid skewing is a non-HSC-autonomous aging feature in HGPS. To assess this possibility, we transplanted CD45.2 BM cells from *Lmna*$^{G609G/G609G}$ mice into lethally irradiated CD45.1 WT recipients. Sixteen weeks later, WT recipients carrying the *Lmna*$^{G609G/G609G}$ mutation only in hematopoietic cells (Figures 6K and 6L) did not reproduce the myeloid skewing and increased platelet counts observed in constitutive *Lmna*$^{G609G/G609G}$ mice (Figures 6C and 6D). Together, these results suggest that premature aging in HGPS affects the hematopoietic system but cannot be explained by HSC-autonomous alterations. Consistent with this idea, microenvironmental alterations (such as sinusoidal vasodilation) were found in *Lmna*$^{G609G/G609G}$ BM (Figures S5D and S5E). Moreover, myelopoietic cytokines augmented in normally aged BM microenvironment (Figures 1J–1N) were also increased in adult progeroid mice (Figures 6M–6Q).

These results suggested that similar microenvironmental alterations might promote myeloid differentiation during physiological/pathological aging. Therefore, we tested whether targeting the microenvironment could impact hematopoiesis in HGPS. Progeroid mice were chronically treated with a β_3-AR agonist, which can rescue nestin$^+$ niches in humans and mice with age-related myeloproliferative disorders (Arranz et al., 2014; Drexler et al., 2019) and has been recently suggested to rejuvenate normally aged HSCs (Maryanovich et al., 2018). Treatment with β_3-AR agonist over 2 months normalized circulating granulocytes and lymphocytes and BM neutrophils and partially rescued BM B cells (Figures 7A–7D). This effect correlated with decreased frequency of BM LT-HSCs (Figures 7E and 7F).

Megakaryocytes have been shown to regulate HSC proliferation (Bruns et al., 2014; Nakamura-Ishizu et al., 2015; Zhao et al., 2014) and their numbers were found to increase during normal/pathological aging in this study. Therefore, we examined the spatial relationships between megakaryocytes and HSPCs in the different models. Like in aged mice (Maryanovich et al., 2018), HSCs expanded but located further from megakaryocytes in adult *Adrb3*$^{-/-}$mice, as an additional premature aging feature in these mice (Figures S6A–S6D). Increased LT-HSC frequency in vehicle-treated progeroid mice (compared with WT mice) (Figures 7E and 7F) correlated with reduced co-localization of HSPCs and megakaryocytes, which was rescued by β_3-AR agonist (Figures 7G–7J). HSCs located more distantly from megakaryocytes in vehicle-treated progeroid mice (compared with WT mice) but this association was partially restored by β_3-AR agonist (Figures 7K–7N). These results suggest the possibility that decreased interactions between megakaryocytes and HSCs might reversibly regulate hematopoiesis during aging.

DISCUSSION

This study suggests that alterations of the BM microenvironment during pathological/physiological aging change hematopoiesis. Remodeling of BM HSC niches (namely reduced endosteal niches and increased non-endosteal neurovascular niches) is associated with the overproduction of pro-inflammatory cytokines which drive excessive myelopoiesis in normal/premature aging. However, the latter can be improved by modulating the microenvironment. Together, these results expand previous findings on the microenvironmental

contribution to hematopoietic aging to the role of different neurovascular beds in normal and pathological aging, and their potential therapeutic targeting.

Stem cell aging compromises tissue turnover and regeneration, but the contribution of the aged stem cell niche remains incompletely understood (López-Otín et al., 2013). In the hematopoietic system, HSCs expand during aging but exhibit impaired self-renewal and lymphoid differentiation potential, favoring myeloid output (Liang et al., 2005; Mohrin et al., 2015; Pang et al., 2011; Rossi et al., 2005; Rundberg Nilsson et al., 2016; Sudo et al., 2000). Moreover, myeloid malignancies are more frequent in the elderly, but whether changes in the aged HSCs and/or their microenvironment predispose to these malignancies has remained unclear. Although HSC aging was initially regarded as solely derived from intrinsic changes in HSCs (Chambers et al., 2007; Dykstra et al., 2011; Florian et al., 2013; Rossi et al., 2007), several alterations in the BM microenvironment or in its interaction with HSCs have been proposed to promote hematopoietic aging (Ergen et al., 2012; Ju et al., 2007; Köhler et al., 2009; Maryanovich et al., 2018). Our study supports this contention by demonstrating that changes in the microenvironment contribute to physiological hematopoietic aging and pathological premature aging (HGPS). In both settings, the aged microenvironment promotes myeloid differentiation through similar cytokines, such as IL-1β and IL-6.

Progeroid mice exhibit several hallmarks of hematopoietic aging: expanded HSCs with reduced engraftment, lymphoid deficiency, and myeloid skewing toward platelets. However, chimeric mice carrying *Lmna*$^{G609G/G609G}$ hematopoietic cells in a WT environment do not reproduce the hematopoietic aging of progeroid mice. Together with the altered vasculature and increased concentration of myelopoietic cytokines in progeroid BM, these results strongly suggest that the BM microenvironment causes premature hematopoietic aging in HGPS. In fact, chronic treatment of progeroid mice with β_3-AR agonist (targeting the microenvironment) reduces HSCs and restores their proximity to megakaryocytes and their lympho-myeloid skewing.

During physiological aging, endosteal BM niches decrease, consistent with previous observations (Kusumbe et al., 2016), whereas non-endosteal neurovascular BM niches containing Nes-GFP$^+$ stromal cells show a pronounced expansion. This microenvironmental remodeling might directly favor myeloid expansion during aging, because lymphoid niches have been found near bone (Morrison and Scadden, 2014), whereas myeloid cell expansion and thrombopoiesis mainly occurs in non-endosteal niches (Eto and Kunishima, 2016).

Recently, an age-related reduction of BM adrenergic nerve fibers has been reported to dive most hallmarks of HSC aging (Maryanovich et al., 2018). However, studies on BM innervation during aging have provided conflicting results, with BM adrenergic fibers reportedly been dramatically reduced (Maryanovich et al., 2018), unchanged, or increased (Chartier et al., 2018). The whole-mount or thick section imaging and 3D reconstruction of different bones in the present study supports the latter and demonstrates that these fibers are doubled in different bones during physiological aging. Moreover, these fibers are not reduced in adult progeroid mice (Figure S7), which exhibit premature hematopoietic aging. Our findings are consistent with the well-known increase in sympathetic activity in the elderly (Hart and Charkoudian, 2014; Ng et al., 1993; Veith et al., 1986; Ziegler et al., 1976) and suggest a functional change of neurotransmission (β_2-AR overriding β_3-AR), rather than a general decline of BM innervation during aging. Actually, increased noradrenergic activity in the BM seems to account for augmented thrombopoiesis in aged mice because platelets are not elevated in aged *Adrb2*$^{-/-}$ mice or *Adrb2*$^{-/-}$*Adrb3*$^{-/-}$ mice. A previous study showed direct effects of α-ARs on mature megakaryocyte adhesion, proplatelet formation, and platelet release, whereas α-AR signaling on more primitive CD34$^+$ progenitor cells did not affect lineage commitment (Chen et al., 2016). However, the present study indicates that HSPC lineage commitment is regulated by the microenvironment through β_2-AR and β_3-AR. Therefore, BM noradrenergic activity appears to regulate lineage commitment and megakaryopoiesis at different stages of maturation through distinct α- and β-ARs. Interestingly, β_2-AR and β_3-AR exhibit opposite roles on myeloid differentiation: whereas β_2-AR signaling promotes megakaryopoiesis through stromal-cell-derived IL6 and becomes predominant during aging, β_3-AR inhibits myelopoiesis. Lack of β_2-AR in the microenvironment (but not in the hematopoietic system) halves myeloid and megakaryocyte progenitors in adult mice. Decreased frequency of megakaryocyte lineage cells is similarly found in adult *Adrb2*$^{-/-}$ mice and aged *Adrb2*$^{-/-}$*Adrb3*$^{-/-}$ mice (but not in WT chimeras carrying *Adrb2*$^{-/-}$ hematopoietic cells), suggesting that predominant β_2-AR activation promotes megakaryocyte differentiation during aging. Whereas adult *Adrb2*$^{-/-}$ mice and *Adrb3*$^{-/-}$ mice exhibit opposite deregulation of lymphoid/myeloid lineage output, only *Adrb2*$^{-/-}$ mice retain this phenotype upon aging (Figures 4B, 4C, and 5A), further suggesting that β_2-AR prevails over β_3-AR in hematopoietic lineage regulation during aging. This regulation requires IL-6, a cytokine known to increase in elderly humans (Brusnahan et al., 2010) and to be similarly regulated in other cell types (Li et al., 2013). Supporting the role of IL6, megakaryocytes appear decreased in *Il6*$^{-/-}$ BM. Moreover, β_2-AR agonist specifically increased IL-6 expression in stromal cells co-cultured with human/murine HSPCs and consequently stimulates megakaryopoiesis in these co-cultures or in primary BM cultures from WT mice, but not *Il6*$^{-/-}$ mice.

In contrast, microenvironmental (but not hematopoietic) lack of β_3-AR partially reduces lymphoid-biased HSCs in adult mice, as previously reported (Maryanovich et al., 2018). However, the results here differ in that β_3-AR loss does not appear overall sufficient to cause premature hematopoietic aging, because the frequencies of circulating lymphocytes and myeloid cells appeared unchanged in 5-month-old *Adrb3*$^{-/-}$ mice. *In vitro*, β_3-AR agonist-treated stromal cells decrease human and murine HSPC differentiation into megakaryocytes. This effect requires Nos1, because *Nos1*$^{-/-}$ mice have high circulating platelets and granulocytes, but low circulating lymphocytes. Furthermore, β_3-AR agonist specifically increases lymphoid-biased HSCs in primary BM cultures in a Nos1-dependent manner. Moreover, adult *Adrb3*$^{-/-}$ or *Nos1*$^{-/-}$ mice share premature microenvironmental aging features in the BM, such as reduced bone-forming transition zone vessels (Kusumbe et al., 2016) and increased BM capillaries, myeloid progenitors, and megakaryocyte apposition to blood vessels.

Megakaryocytes inhibit HSC proliferation (Bruns et al., 2014; Nakamura-Ishizu et al., 2015; Zhao et al., 2014). Whereas both

HSCs and megakaryocytes expand during aging, HSCs locate further from megakaryocytes in adult *Adrb3*$^{-/-}$ mice and progeroid mice, suggesting that decreased HSC-megakaryocyte interactions might contribute to premature hematopoietic aging features in these mice. Supporting this possibility, chronic treatment of progeroid mice with β_3-AR agonist decreased HSCs and corrected lineage skewing, correlated with normalized distribution of HSCs near megakaryocytes. Future studies will be required to investigate changes in the regulation of HSC proliferation by megakaryocytes during aging.

In summary, this study shows that the aged BM microenvironment promotes myeloid expansion during physiological aging and in premature aging. Normal murine aging concurs with the reduction of endosteal niches and the expansion of non-endosteal niches comprising capillaries and nestin$^+$ cells associated with sympathetic noradrenergic fibers. Interestingly, β_2-AR and β_3-AR regulate myelopoiesis through opposite and stage-dependent effects on the hematopoietic microenvironment. During normal aging, increased β_2-AR activity promotes IL6-dependent myeloid differentiation, whereas decreased β_3-AR-Nos1-NO is associated with reduced endosteal niches and increased central niches. Adult Nos1 KO mice and progeroid mice display premature aging in peripheral blood, manifested as reduced lymphocytes and increased myeloid cells. Megakaryocytes move closer to BM sinusoids in *Adrb3*$^{-/-}$ or progeroid mice, possibly explaining increased thrombopoiesis. Megakaryocytes and HSCs expand but separate from each other during normal aging and in adult *Adrb3*$^{-/-}$ or progeroid mice. However, chronic treatment of progeroid mice with β_3-AR agonist reduces HSCs and restores their proximity to megakaryocytes and their lympho-myeloid skewing. Therefore, normal or premature niche aging promotes normal myeloid expansion, which can be improved by targeting the microenvironment.

STAR★METHODS

Detailed methods are provided in the online version of this paper and include the following:

- KEY RESOURCES TABLE
- LEAD CONTACT AND MATERIALS AVAILABILITY
- METHODS DETAILS
  - Mouse strains
  - Mouse bone marrow transplantation and *in vivo* treatments
  - BM cell extraction, flow cytometry and fluorescence-activated cell sorting
  - Cell culture
  - Immunofluorescence staining
  - RNA isolation and qPCR
  - ELISA
  - Measurement of nitrate concentration
  - Statistical analyses
- DATA AND CODE AVAILABILITY

SUPPLEMENTAL INFORMATION

Supplemental Information can be found online at https://doi.org/10.1016/j.stem.2019.06.007.

ACKNOWLEDGMENTS

We thank A.R. Green for advice and support; M. García-Fernández, C. Fielding, C. Kapeni, X. Langa, and other current and former members of the S.M.-F. group for help and discussions; A. Barettino and A. Macías (CNIC), D. Pask, T. Hamilton, the Central Biomedical Services and Cambridge NIHR BRC Cell Phenotyping Hub for technical assistance; and H. Jolin and A.N.J. McZenzie (MRC Laboratory of Molecular Biology, Cambridge, UK) for help with milliplex analyses. Y.-H.O. received fellowships from Alborada Scholarship (University of Cambridge), Trinity-Henry Barlow Scholarship (University of Cambridge), and R.O.C. Government Scholarship to Study Abroad (GSSA). A.G.G. received fellowships from the Ramón Areces Foundation and the LaCaixa Foundation. C.K. was supported by Marie Curie Career Integration (H2020-MSCA-IF-2015-70841). S.M.-F. was supported by Red TerCel (ISCIII-Spanish Cell Therapy Network). V.A. is supported by grants from the Spanish Ministerio de Economía, Industria y Competitividad (MEIC) with co-funding from the Fondo Europeo de Desarrollo Regional (FEDER, "Una manera de hacer Europa") (SAF2016-79490-R), the Instituto de Salud Carlos III (AC16/00091 and AC17/00067), the Fundació Marató TV3 (122/C/2015), and the Progeria Research Foundation (Established Investigator Award 2014–52). The CNIC is supported by the Instituto de Salud Carlos III (ISCIII), the Ministerio de Ciencia, Innovación y Universidades (MCIU), and the Pro CNIC Foundation, and is a Severo Ochoa Center of Excellence (SEV-2015-0505). This work was supported by core support grants from the Wellcome Trust and the MRC to the Cambridge Stem Cell Institute, MEIC (SAF-2011-30308), Ramón y Cajal Program Grant (RYC-2009-04703), ConSEPOC-Comunidad de Madrid (S2010/BMD-2542), National Health Service Blood and Transplant (United Kingdom), European Union's Horizon 2020 research (ERC-2014-CoG-64765 and Marie Curie Career Integration grant FP7-PEOPLE-2011-RG-294096), and a Programme Foundation Award from Cancer Research UK to S.M.-F., who was also supported in part by an International Early Career Scientist grant from the Howard Hughes Medical Institute.

AUTHOR CONTRIBUTIONS

Y.-H.H., R.d.T., J.R.-T., J.R., C.K., A.G.-G., D.M., A.d.M., and C.G.-G. designed and performed experiments and analyzed data. A.K.W. and H.R.F. assisted with co-culture experiments. M.W. and F.L analyzed *Nos1* KO mice. C.L.-O. provided progeroid mice. R.S.J. facilitated nitrate measurements. C.N. provided *Vwf-eGFP* mice. W.V., C.G., and V.A. provided advise on the design, supervision, and analysis of experiments. Y.-H.H. and S.M.-F. prepared the figures and wrote the manuscript. S.M.-F. planned and supervised the overall study. All authors revised and approved the manuscript.

DECLARATION OF INTERESTS

The authors declare no competing interests.

Received: July 30, 2018
Revised: February 21, 2019
Accepted: June 10, 2019
Published: July 11, 2019

REFERENCES

Arranz, L., Sánchez-Aguilera, A., Martín-Pérez, D., Isern, J., Langa, X., Tzankov, A., Lundberg, P., Muntión, S., Tzeng, Y.S., Lai, D.M., et al. (2014). Neuropathy of haematopoietic stem cell niche is essential for myeloproliferative neoplasms. Nature *512*, 78–81.

Bruns, I., Lucas, D., Pinho, S., Ahmed, J., Lambert, M.P., Kunisaki, Y., Scheiermann, C., Schiff, L., Poncz, M., Bergman, A., and Frenette, P.S. (2014). Megakaryocytes regulate hematopoietic stem cell quiescence through CXCL4 secretion. Nat. Med. *20*, 1315–1320.

Brusnahan, S.K., McGuire, T.R., Jackson, J.D., Lane, J.T., Garvin, K.L., O'Kane, B.J., Berger, A.M., Tuljapurkar, S.R., Kessinger, M.A., and Sharp, J.G. (2010). Human blood and marrow side population stem cell and Stro-1 positive bone marrow stromal cell numbers decline with age, with an increase

in quality of surviving stem cells: correlation with cytokines. Mech. Ageing Dev. *131*, 718–722.

Chambers, S.M., Shaw, C.A., Gatza, C., Fisk, C.J., Donehower, L.A., and Goodell, M.A. (2007). Aging hematopoietic stem cells decline in function and exhibit epigenetic dysregulation. PLoS Biol. *5*, e201.

Chartier, S.R., Mitchell, S.A.T., Majuta, L.A., and Mantyh, P.W. (2018). The Changing Sensory and Sympathetic Innervation of the Young, Adult and Aging Mouse Femur. Neuroscience *387*, 178–190.

Chen, S., Du, C., Shen, M., Zhao, G., Xu, Y., Yang, K., Wang, X., Li, F., Zeng, D., Chen, F., et al. (2016). Sympathetic stimulation facilitates thrombopoiesis by promoting megakaryocyte adhesion, migration, and proplatelet formation. Blood *127*, 1024–1035.

Chruscinski, A.J., Rohrer, D.K., Schauble, E., Desai, K.H., Bernstein, D., and Kobilka, B.K. (1999). Targeted disruption of the beta2 adrenergic receptor gene. J. Biol. Chem. *274*, 16694–16700.

De Sandre-Giovannoli, A., Bernard, R., Cau, P., Navarro, C., Amiel, J., Boccaccio, I., Lyonnet, S., Stewart, C.L., Munnich, A., Le Merrer, M., and Lévy, N. (2003). Lamin a truncation in Hutchinson-Gilford progeria. Science *300*, 2055.

Drexler, B., Passweg, J.R., Tzankov, A., Bigler, M., Theocharides, A.P.A., Cantoni, N., Keller, P., Stussi, G., Ruefer, A., Benz, R., et al. (2019). The sympathomimetic agonist mirabegron did not lower JAK2-V617F allele burden, but restored nestin-positive cells and reduced reticulin fibrosis in patients with myeloproliferative neoplasms: results of phase 2 study SAKK 33/14. Haematologica *104*, 710–716.

Dykstra, B., Olthof, S., Schreuder, J., Ritsema, M., and de Haan, G. (2011). Clonal analysis reveals multiple functional defects of aged murine hematopoietic stem cells. J. Exp. Med. *208*, 2691–2703.

Elefteriou, F., Ahn, J.D., Takeda, S., Starbuck, M., Yang, X., Liu, X., Kondo, H., Richards, W.G., Bannon, T.W., Noda, M., et al. (2005). Leptin regulation of bone resorption by the sympathetic nervous system and CART. Nature *434*, 514–520.

Ergen, A.V., Boles, N.C., and Goodell, M.A. (2012). Rantes/Ccl5 influences hematopoietic stem cell subtypes and causes myeloid skewing. Blood *119*, 2500–2509.

Eriksson, M., Brown, W.T., Gordon, L.B., Glynn, M.W., Singer, J., Scott, L., Erdos, M.R., Robbins, C.M., Moses, T.Y., Berglund, P., et al. (2003). Recurrent de novo point mutations in lamin A cause Hutchinson-Gilford progeria syndrome. Nature *423*, 293–298.

Eto, K., and Kunishima, S. (2016). Linkage between the mechanisms of thrombocytopenia and thrombopoiesis. Blood *127*, 1234–1241.

Flach, J., Bakker, S.T., Mohrin, M., Conroy, P.C., Pietras, E.M., Reynaud, D., Alvarez, S., Diolaiti, M.E., Ugarte, F., Forsberg, E.C., et al. (2014). Replication stress is a potent driver of functional decline in ageing haematopoietic stem cells. Nature *512*, 198–202.

Florian, M.C., Nattamai, K.J., Dörr, K., Marka, G., Uberle, B., Vas, V., Eckl, C., Andrä, I., Schiemann, M., Oostendorp, R.A., et al. (2013). A canonical to non-canonical Wnt signalling switch in haematopoietic stem-cell ageing. Nature *503*, 392–396.

Gauthier, C., Leblais, V., Kobzik, L., Trochu, J.N., Khandoudi, N., Bril, A., Balligand, J.L., and Le Marec, H. (1998). The negative inotropic effect of beta3-adrenoceptor stimulation is mediated by activation of a nitric oxide synthase pathway in human ventricle. J. Clin. Invest. *102*, 1377–1384.

Gekas, C., and Graf, T. (2013). CD41 expression marks myeloid-biased adult hematopoietic stem cells and increases with age. Blood *121*, 4463–4472.

Grigoryan, A., Guidi, N., Senger, K., Liehr, T., Soller, K., Marka, G., Vollmer, A., Markaki, Y., Leonhardt, H., Buske, C., et al. (2018). LaminA/C regulates epigenetic and chromatin architecture changes upon aging of hematopoietic stem cells. Genome Biol. *19*, 189.

Grover, A., Sanjuan-Pla, A., Thongjuea, S., Carrelha, J., Giustacchini, A., Gambardella, A., Macaulay, I., Mancini, E., Luis, T.C., Mead, A., et al. (2016). Single-cell RNA sequencing reveals molecular and functional platelet bias of aged haematopoietic stem cells. Nat. Commun. *7*, 11075.

Hamczyk, M.R., Villa-Bellosta, R., Gonzalo, P., Andrés-Manzano, M.J., Nogales, P., Bentzon, J.F., López-Otín, C., and Andrés, V. (2018). Vascular Smooth Muscle-Specific Progerin Expression Accelerates Atherosclerosis and Death in a Mouse Model of Hutchinson-Gilford Progeria Syndrome. Circulation *138*, 266–282.

Hart, E.C., and Charkoudian, N. (2014). Sympathetic neural regulation of blood pressure: influences of sex and aging. Physiology (Bethesda) *29*, 8–15.

Ho, T.T., Warr, M.R., Adelman, E.R., Lansinger, O.M., Flach, J., Verovskaya, E.V., Figueroa, M.E., and Passegué, E. (2017). Autophagy maintains the metabolism and function of young and old stem cells. Nature *543*, 205–210.

Huang, P.L., Dawson, T.M., Bredt, D.S., Snyder, S.H., and Fishman, M.C. (1993). Targeted disruption of the neuronal nitric oxide synthase gene. Cell *75*, 1273–1286.

Isern, J., García-García, A., Martín, A.M., Arranz, L., Martín-Pérez, D., Torroja, C., Sánchez-Cabo, F., and Méndez-Ferrer, S. (2014). The neural crest is a source of mesenchymal stem cells with specialized hematopoietic stem cell niche function. eLife *3*, e03696.

Ju, Z., Jiang, H., Jaworski, M., Rathinam, C., Gompf, A., Klein, C., Trumpp, A., and Rudolph, K.L. (2007). Telomere dysfunction induces environmental alterations limiting hematopoietic stem cell function and engraftment. Nat. Med. *13*, 742–747.

Köhler, A., Schmithorst, V., Filippi, M.D., Ryan, M.A., Daria, D., Gunzer, M., and Geiger, H. (2009). Altered cellular dynamics and endosteal location of aged early hematopoietic progenitor cells revealed by time-lapse intravital imaging in long bones. Blood *114*, 290–298.

Kopf, M., Baumann, H., Freer, G., Freudenberg, M., Lamers, M., Kishimoto, T., Zinkernagel, R., Bluethmann, H., and Köhler, G. (1994). Impaired immune and acute-phase responses in interleukin-6-deficient mice. Nature *368*, 339–342.

Kusumbe, A.P., Ramasamy, S.K., Itkin, T., Mäe, M.A., Langen, U.H., Betsholtz, C., Lapidot, T., and Adams, R.H. (2016). Age-dependent modulation of vascular niches for haematopoietic stem cells. Nature *532*, 380–384.

Li, W., Shi, X., Wang, L., Guo, T., Wei, T., Cheng, K., Rice, K.C., Kingery, W.S., and Clark, J.D. (2013). Epidermal adrenergic signaling contributes to inflammation and pain sensitization in a rat model of complex regional pain syndrome. Pain *154*, 1224–1236.

Liang, Y., Van Zant, G., and Szilvassy, S.J. (2005). Effects of aging on the homing and engraftment of murine hematopoietic stem and progenitor cells. Blood *106*, 1479–1487.

López-Otín, C., Blasco, M.A., Partridge, L., Serrano, M., and Kroemer, G. (2013). The hallmarks of aging. Cell *153*, 1194–1217.

Maryanovich, M., Zahalka, A.H., Pierce, H., Pinho, S., Nakahara, F., Asada, N., Wei, Q., Wang, X., Ciero, P., Xu, J., et al. (2018). Adrenergic nerve degeneration in bone marrow drives aging of the hematopoietic stem cell niche. Nat. Med. *24*, 782–791.

Méndez-Ferrer, S., Lucas, D., Battista, M., and Frenette, P.S. (2008). Haematopoietic stem cell release is regulated by circadian oscillations. Nature *452*, 442–447.

Méndez-Ferrer, S., Battista, M., and Frenette, P.S. (2010a). Cooperation of beta(2)- and beta(3)-adrenergic receptors in hematopoietic progenitor cell mobilization. Ann. N Y Acad. Sci. *1192*, 139–144.

Méndez-Ferrer, S., Michurina, T.V., Ferraro, F., Mazloom, A.R., Macarthur, B.D., Lira, S.A., Scadden, D.T., Ma'ayan, A., Enikolopov, G.N., and Frenette, P.S. (2010b). Mesenchymal and haematopoietic stem cells form a unique bone marrow niche. Nature *466*, 829–834.

Merideth, M.A., Gordon, L.B., Clauss, S., Sachdev, V., Smith, A.C., Perry, M.B., Brewer, C.C., Zalewski, C., Kim, H.J., Solomon, B., et al. (2008). Phenotype and course of Hutchinson-Gilford progeria syndrome. N. Engl. J. Med. *358*, 592–604.

Mignone, J.L., Kukekov, V., Chiang, A.S., Steindler, D., and Enikolopov, G. (2004). Neural stem and progenitor cells in nestin-GFP transgenic mice. J. Comp. Neurol. *469*, 311–324.

Mohrin, M., Shin, J., Liu, Y., Brown, K., Luo, H., Xi, Y., Haynes, C.M., and Chen, D. (2015). Stem cell aging. A mitochondrial UPR-mediated metabolic checkpoint regulates hematopoietic stem cell aging. Science *347*, 1374–1377.

Morrison, S.J., and Scadden, D.T. (2014). The bone marrow niche for haematopoietic stem cells. Nature *505*, 327–334.

Nakamura-Ishizu, A., Takubo, K., Kobayashi, H., Suzuki-Inoue, K., and Suda, T. (2015). CLEC-2 in megakaryocytes is critical for maintenance of hematopoietic stem cells in the bone marrow. J. Exp. Med. *212*, 2133–2146.

Ng, A.V., Callister, R., Johnson, D.G., and Seals, D.R. (1993). Age and gender influence muscle sympathetic nerve activity at rest in healthy humans. Hypertension *21*, 498–503.

Osorio, F.G., Navarro, C.L., Cadiñanos, J., López-Mejía, I.C., Quirós, P.M., Bartoli, C., Rivera, J., Tazi, J., Guzmán, G., Varela, I., et al. (2011). Splicing-directed therapy in a new mouse model of human accelerated aging. Sci. Transl. Med. *3*, 106ra107.

Pang, W.W., Price, E.A., Sahoo, D., Beerman, I., Maloney, W.J., Rossi, D.J., Schrier, S.L., and Weissman, I.L. (2011). Human bone marrow hematopoietic stem cells are increased in frequency and myeloid-biased with age. Proc. Natl. Acad. Sci. USA *108*, 20012–20017.

Pietras, E.M. (2017). Inflammation: a key regulator of hematopoietic stem cell fate in health and disease. Blood *130*, 1693–1698.

Pinho, S., Marchand, T., Yang, E., Wei, Q., Nerlov, C., and Frenette, P.S. (2018). Lineage-Biased Hematopoietic Stem Cells Are Regulated by Distinct Niches. Dev. Cell *44*, 634–641.

Pinto do O, P., Kolterud, A., and Carlsson, L. (1998). Expression of the LIM-homeobox gene LH2 generates immortalized steel factor-dependent multipotent hematopoietic precursors. EMBO J. *17*, 5744–5756.

Plo, I., Bellanné-Chantelot, C., Mosca, M., Mazzi, S., Marty, C., and Vainchenker, W. (2017). Genetic Alterations of the Thrombopoietin/MPL/JAK2 Axis Impacting Megakaryopoiesis. Front. Endocrinol. (Lausanne) *8*, 234.

Rosenbaum, D.M., Rasmussen, S.G., and Kobilka, B.K. (2009). The structure and function of G-protein-coupled receptors. Nature *459*, 356–363.

Rossi, D.J., Bryder, D., Zahn, J.M., Ahlenius, H., Sonu, R., Wagers, A.J., and Weissman, I.L. (2005). Cell intrinsic alterations underlie hematopoietic stem cell aging. Proc. Natl. Acad. Sci. USA *102*, 9194–9199.

Rossi, D.J., Bryder, D., Seita, J., Nussenzweig, A., Hoeijmakers, J., and Weissman, I.L. (2007). Deficiencies in DNA damage repair limit the function of haematopoietic stem cells with age. Nature *447*, 725–729.

Rundberg Nilsson, A., Soneji, S., Adolfsson, S., Bryder, D., and Pronk, C.J. (2016). Human and Murine Hematopoietic Stem Cell Aging Is Associated with Functional Impairments and Intrinsic Megakaryocytic/Erythroid Bias. PLoS ONE *11*, e0158369.

Sanjuan-Pla, A., Macaulay, I.C., Jensen, C.T., Woll, P.S., Luis, T.C., Mead, A., Moore, S., Carella, C., Matsuoka, S., Bouriez Jones, T., et al. (2013). Platelet-biased stem cells reside at the apex of the haematopoietic stem-cell hierarchy. Nature *502*, 232–236.

Stegner, D., vanEeuwijk, J.M.M., Angay, O., Gorelashvili, M.G., Semeniak, D., Pinnecker, J., Schmithausen, P., Meyer, I., Friedrich, M., Dütting, S., et al. (2017). Thrombopoiesis is spatially regulated by the bone marrow vasculature. Nat. Commun. *8*, 127.

Sudo, K., Ema, H., Morita, Y., and Nakauchi, H. (2000). Age-associated characteristics of murine hematopoietic stem cells. J. Exp. Med. *192*, 1273–1280.

Susulic, V.S., Frederich, R.C., Lawitts, J., Tozzo, E., Kahn, B.B., Harper, M.E., Himms-Hagen, J., Flier, J.S., and Lowell, B.B. (1995). Targeted disruption of the beta 3-adrenergic receptor gene. J. Biol. Chem. *270*, 29483–29492.

Takeda, S., Elefteriou, F., Levasseur, R., Liu, X., Zhao, L., Parker, K.L., Armstrong, D., Ducy, P., and Karsenty, G. (2002). Leptin regulates bone formation via the sympathetic nervous system. Cell *111*, 305–317.

Veith, R.C., Featherstone, J.A., Linares, O.A., and Halter, J.B. (1986). Age differences in plasma norepinephrine kinetics in humans. J. Gerontol. *41*, 319–324.

Villa-Bellosta, R., Rivera-Torres, J., Osorio, F.G., Acín-Pérez, R., Enriquez, J.A., López-Otín, C., and Andrés, V. (2013). Defective extracellular pyrophosphate metabolism promotes vascular calcification in a mouse model of Hutchinson-Gilford progeria syndrome that is ameliorated on pyrophosphate treatment. Circulation *127*, 2442–2451.

Yamamoto, R., Morita, Y., Ooehara, J., Hamanaka, S., Onodera, M., Rudolph, K.L., Ema, H., and Nakauchi, H. (2013). Clonal analysis unveils self-renewing lineage-restricted progenitors generated directly from hematopoietic stem cells. Cell *154*, 1112–1126.

Yirmiya, R., Goshen, I., Bajayo, A., Kreisel, T., Feldman, S., Tam, J., Trembovler, V., Csernus, V., Shohami, E., and Bab, I. (2006). Depression induces bone loss through stimulation of the sympathetic nervous system. Proc. Natl. Acad. Sci. USA *103*, 16876–16881.

Zhao, M., Perry, J.M., Marshall, H., Venkatraman, A., Qian, P., He, X.C., Ahamed, J., and Li, L. (2014). Megakaryocytes maintain homeostatic quiescence and promote post-injury regeneration of hematopoietic stem cells. Nat. Med. *20*, 1321–1326.

Ziegler, M.G., Lake, C.R., and Kopin, I.J. (1976). Plasma noradrenaline increases with age. Nature *261*, 333–335.

STAR★METHODS

KEY RESOURCES TABLE

REAGENT or RESOURCE	SOURCE	IDENTIFIER
Antibodies		
CD45.1 (A20)	BD Biosciences	Cat#560578; RRID:AB_1727488
CD45.2 (104)	TONBO Biosciences	Cat#20-0454-U100; RRID:AB_2621576
B220 (RA3-6B2)	BD Biosciences	Cat#553088; RRID:AB_394618
CD11b (M1/70)	BioLegend	Cat#101208; RRID:AB_312791
CD3ε (145-2C11)	TONBO Biosciences	Cat#65-0031-U100; RRID:AB_2621872
Ly-6G (RB6-(C5)	BioLegend	Cat#127623; RRID:AB_10645331
CD90 (53-2.1)	BD Biosciences	Cat#553003; RRID:AB_394542
sca-1 (E13-161.7)	BioLegend	Cat#108128; RRID:AB_2563064
CD42d (1C2)	Fisher Scientific Ltd	Cat#17-0421-80; RRID:AB_1724073
biotinylated lineage antibodies (CD11b, Gr-1, Ter119, B220, CD3ε)	BD Biosciences	Cat#559971; RRID:AB_10053179
c-kit (2B8)	Thermo Fisher Scientific	Cat#11-1171-81; RRID:AB_465185
CD150 (TC15-12F12.2)	BioLegend	Cat#115927; RRID:AB_11204248
CD34 (RAM34)	BD Biosciences	Cat#560238; RRID:AB_1645242
CD41 (MWReg30)	BioLegend	Cat#133905; RRID:AB_2265179
Brilliant Violet 510 Streptavidin	BioLegend	Cat#405233
anti-CD45-biotinylated antibody	BD Biosciences	Cat#553078; RRID:AB_394608
anti-Ter119 –biotinylated antibody	BD Biosciences	Cat#553672; RRID:AB_394985
anti-CD31-APC antibody	BD Biosciences	Cat#551262; RRID:AB_398497
Streptavidin-APCCy7	BD Biosciences	Cat#554063; RRID:AB_10054651
anti-ckit-FITC antibody	BD PharMingen	Cat#553354; RRID:AB_394805
anti-CD41-PE antibody	BD PharMingen	Cat#558040; RRID:AB_397004
Human/Mouse CD117/c-kit Antibody (Polyclonal Goat IgG)	R&D	Cat#AF1356; RRID:AB_354750
rat anti-CD150 antibody	Biolegend	Cat#115902; RRID:AB_313681
CD34 Microbead kit	Miltenyi Biotec	Cat#130-046-702
rabbit anti-TH antibody	Millipore	Cat#AB152; RRID:AB_390204
Alexa Fluor 647 goat anti-rat IgG	Life Technologies	Cat#A21247; RRID:AB_141778
anti-rabbit biotinylated antibody	Jackson ImmunoResearch Labs	Cat#111-066-003; RRID:AB_2337966
Cy3-Tyramide secondary antibody	Perkin Elmer	Cat#SAT704A001EA
rat anti-EMCN antibody	Santa Cruz Biotechnology	Cat#sc-65495; RRID:AB_2100037
goat anti-CD31 antibody	R&D	Cat#AF3628; RRID:AB_2161028
Alexa Fluor 546 Donkey anti-rabbit IgG	Thermo Fisher Scientific	Cat#A10040; RRID:AB_2534016
Dylight650 donkey anti-rat IgG	Thermo Fisher Scientific	Cat#SA5-10029; RRID:AB_2556609
Cy3-donkey anti-goat IgG	Jackson ImmunoResearch Labs	Cat#705-165-147; RRID:AB_2307351
Alexa Fluor 647 donkey-anti-goat IgG	Life Technologies	Cat#A21447; RRID:AB_141844
Alexa Fluor 647 Goat-anti-armenian hamster IgG	Abcam	Cat#ab173004; RRID:AB_2732023
mouse-anti-human CD61 primary antibody	Serotec	Cat#MCA728; RRID:AB_321515
Alexa Fluor 546 donkey-anti-mouse IgG	Thermo Fisher Scientific	Cat#A10036; RRID:AB_2534012
Alexa Fluor 488 Sreptavidin-conjugated antibody	Thermo Fisher Scientific	Cat#S32354; RRID:AB_2315383
Alexa Fluor 555 goat-anti-rat IgG	Thermo Fisher Scientific	Cat#A21434; RRID:AB_2535855

(*Continued on next page*)

Continued

REAGENT or RESOURCE	SOURCE	IDENTIFIER
Chemicals, Peptides, and Recombinant Proteins		
Trizol Reagent	Sigma-Aldrich	Cat#T9424
α-MEM medium	StemCell Technologies	Cat#36450
MyeloCult M5300 medium	StemCell Technologies	Cat#05350
StemSpan H3000 medium	StemCell Technologies	Cat#09850
IMDM medium	ThermoFisher	Cat#12440053
Ammonium chloride	Sigma-Aldrich	Cat#254134
collagenase type I	Stem Cell Technologies	Cat#07902
DAPI	Sigma Aldrich	Cat#D9542
mSCF	PeProTech	Cat#250-03-100
TPO	PeProTech	Cat#AF-300-18
EPO	R&D	Cat#287-TC-500
IL-1β	Cellgenix	Cat#1411-050
BRL37344	Sigma-Aldrich	Cat#B169
H-89	Sigma-Aldrich	Cat#B1427
Clenbuterol hydrochloride	Sigma-Aldrich	Cat#C5423
hydrocortisone	StemCell Technologies	Cat#07904
Avidin/biotin blocking kit	Vector Laboratories	Cat#SP-2001
Triton X-100	Sigma-Aldrich	Cat#T8787
TNB (0.1 M Tris–HCl, pH7.5, 0.15 M NaCl, 0.5% blocking reagent)	Perkin Elmer	Cat#FP1020
DPX Mountant for histology	Sigma-Aldrich	Cat#44581
donkey serum	Sigma-Aldrich	Cat#D9663
rat serum	Sigma-Aldrich	Cat#R9759
Dako Fluorescence Mounting Medium	Agilent	Cat#S3023
DMSO	Sigma-Aldrich	Cat#D5879
Critical Commercial Assays		
Lympholyte®-M Cell Separation Media	Cedarlane	Cat#CL5031
ABC amplification kit	Vector Labs	Cat#AK-5000
MILLIPLEX MAP Mouse Cytokine/ Chemokine Magnetic Bead Panel	Merck Millipore	Cat#MCYTOMAG-70K
High-Capacity cDNA Reverse Transcription kit	Applied Biosystems	Cat#4368814
PowerUp SYBR Green Master Mix	Applied Biosystems	Cat#A25742
Experimental Models: Organisms/Strains		
Nes-gfp	Prof. Grigori N. Eikolopov, Stony Brook, USA	Mignone et al., 2004
Adrb2$^{-/-}$	Prof. Gerard Karsenty, Columbia University, New York, USA	Chruscinski et al., 1999
FVB/N-Adrb3tm1Lowl/J	The Jackson Laboratory	Stock#6402, backcrossed to C57BL/6J for 10 generations
Lmnatm1.1Otin	Prof. Carlos López-Otín, Oviedo University, Spain	Osorio et al., 2011
Vwf-eGFP	Prof. Claus Nerlov, University of Oxford, UK	Sanjuan-Pla et al., 2013
Nos1tm1Plh/J	The Jackson Laboratory	Stock#2986
Il6tm1Kopf/J	The Jackson Laboratory	Stock#2650
congenic CD45.1 C57BL/6	Charles River Laboratories	N/A
congenic CD45.2 C57BL/6	Charles River Laboratories	N/A

(*Continued on next page*)

Continued		
REAGENT or RESOURCE	SOURCE	IDENTIFIER
Experimental Models: Cell lines		
MS-5	DSMZ	ACC 441
HPC-7	Prof. Leif Carlsson, Umeå University, Sweden	N/A
Oligonucleotides		
NOS1-Fw: ACTGACACCCTGCACCT GAAGA	Sigma-Aldrich	N/A
NOS1-Rv: GTGCGGACATCTTCTGA CTTCC	Sigma-Aldrich	N/A
NOS2-Fw: CAGCTGGGCTGTACAAACCTT	Sigma-Aldrich	N/A
NOS2-Rv: CATTGGAAGTGAAGCGGTTCG	Sigma-Aldrich	N/A
NOS3-Fw: CCTCGAGTAAAGAACTGG GAAGTG	Sigma-Aldrich	N/A
NOS3-Rv: AACTTCCTTGGAAACAC CAGGG	Sigma-Aldrich	N/A
Gapdh-Fw: GCATGGCCTTCCGTGTTC	Sigma-Aldrich	N/A
Gapdh-Rv: CTGCTTCACCACCTTCTTGAT	Sigma-Aldrich	N/A

LEAD CONTACT AND MATERIALS AVAILABILITY

Further information and requests for resources and reagents should be addressed to the Lead Contact, Dr. Simon Mendez-Ferrer, at sm2116@medschl.cam.ac.uk.

METHODS DETAILS

Mouse strains

Young mice were analyzed between 8-30 weeks of age, and old mice were 66-120 weeks old. Mice were housed in specific pathogen free facilities. All experiments using mice followed protocols approved by the Animal Welfare Ethical Committees, according to EU and United Kingdom Home Office regulations (PPL 70/8406). *Nes-gfp* (Mignone et al., 2004), *Adrb2*$^{-/-}$ (Chruscinski et al., 1999), *FVB/N-Adrb3tm1Lowl/J* (Susulic et al., 1995), *Lmnatm1.1Otin* (Osorio et al., 2011), *Vwf-egfp* (Sanjuan-Pla et al., 2013), *B6.129S4-Nos1tm1Plh/J* (stock#2986) (Huang et al., 1993), *B6.129S2-Il6tm1Kopf/J* (stock#2650) (Kopf et al., 1994) (Jackson Laboratories) and congenic CD45.1 and CD45.2 C57BL/6 mice (Charles River Laboratories) were used in this study.

Mouse bone marrow transplantation and *in vivo* treatments

In *Lmna*$^{G609G/G609G}$ mouse model, HSC activity was assessed by long-term competitive repopulation assay using the congenic CD45.1/CD45.2 isotypes. Recipient female CD45.1 C57BL/6 mice (8 week-old) were subjected to lethal irradiation (12 Gy whole body irradiation, split dose 6.0 + 6.0 Gy, 3 h apart). 10^6 BM nucleated cells from (CD45.2) WT mice or LmnaG619G/G609G mice were mixed with 10^6 BM nucleated cells from CD45.1 C57BL/6 mice and i.v. transplanted in 200 µL sterile PBS into lethally irradiated CD45.1 C57BL/6 mice. At various time points after transplantation (4, 8, 12 and 16 weeks), peripheral blood nucleated cells were collected from recipients. Cells were stained with fluorescent-conjugated antibodies against B220, Mac1 and CD90 antigens, and analyzed by fluorescence-activated cell sorting. The selective β3-AR agonist BRL37344 (Sigma, St. Louis, MO) was administered at 2mg/kg through intraperitoneal (i.p.) injection once per day. Vehicle (saline solution) daily injections were performed in the same way. Treatment was initiated when *Lmna*$^{G609G/G609G}$ mice were 7 weeks old and lasted for 8 weeks.

To generate chimeric mice carrying *Adrb2*$^{-/-}$ or *Adrb3*$^{-/-}$ BM cells, two-month-old C57BL/6 mice were lethally irradiated (12 Gy, two split doses) and i.v. transplanted with 2 X 10^6 nucleated BM cells from two-month-old WT, *Adrb2*$^{-/-}$ or *Adrb3*$^{-/-}$ mice. To test microenvironment-dependent effects of β-adrenergic signaling, WT, *Adrb2*$^{-/-}$ or *Adrb3*$^{-/-}$ (CD45.2) mice were lethally irradiated (12 Gy, two split doses) and i.v. transplanted with 2 X 10^6 nucleated BM cells from CD45.1 C57BL/6 mice. Hematopoietic cells in the BM were analyzed 4 months after transplantation in both settings.

BM cell extraction, flow cytometry and fluorescence-activated cell sorting

For BM hematopoietic cell isolation, bones were crushed in a mortar, filtered through a 40-µm strainer to obtain single cell suspensions, and depleted of red blood cells by lysis in 0.15 M NH_4Cl for 10 min at 4°C. Blood samples were directly lysed. Cells were incubated with the appropriate dilution (2-5 µg/ml) of fluorescent antibody conjugates and 4',6-diamidino-2-phenylindole (DAPI) for dead cell exclusion, and analyzed on LSRFortessa flow cytometer (BD Biosciences, Franklin Lakes, NJ) equipped with FACSDiva Software

(BD Biosciences). The following antibodies were used: fluorescent CD45.1 (A20), CD45.2 (104), B220 (RA3-6B2), CD11b (M1/70), CD3ε (145-2C11), Ly-6G (1A8), CD90 (53-2.1) sca-1 (E13-161.7), CD42d (1C2), biotinylated lineage antibodies (CD11b, Gr-1, Ter119, B220, CD3ε) (BD Biosciences), c-kit (2B8) (eBioscience), CD150 (TC15-12F12.2), CD34 (RAM34) and CD41 (MWReg30) (BioLegend). Biotinylated antibodies were detected with fluorochrome conjugated streptavidin (BD Biosciences).

To isolate endosteal and non-endosteal HSPCs, long bones were flushed gently to obtain hematopoietic cells less tightly associated with the bone and then flushed-bones were crushed in a mortar to obtain hematopoietic cells enriched in the endosteal compartment. Cells were stained with the above-mentioned antibodies and analyzed by flow cytometry or sorted (FACS Aria cell sorter, BD Bioscience). Long-term HSCs (LT-HSCs) were immunophenotypically defined as lin-sca-1+ckit+CD34-CD150+CD41- cells. Myeloid-biased HSCs were immunophenotypically defined as lin-sca-1+ckit+CD34-CD150+CD41+ cells. Lymphoid-biased HSCs were immunophenotypically defined as lin-sca-1+ ckit+CD34-CD150-CD41- cells.

For analysis of Nes-GFP+ cell distribution in endosteal and non-endosteal BM fractions, the long bones were flushed with PBS and remaining endosteal part was crushed in a mortar. Both fractions were digested in 2ml of collagenase type I (Stem Cell Technologies, cat. No. 07902) for 30 min at 37°C with agitation. The enzyme was quenched by adding 18ml PBS/2%FCS. Cell suspensions were filtered, pelleted and red blood cell lysis was performed as stated above. Samples were stained with the following antibodies: 1:100 anti-CD45-biotinylated ab (BD Biosciences, cat. No. 553078), 1:100 anti-Ter119 –biotinylated ab (BD Biosciences, cat. No. 553672), 1:100 anti-CD31-APC ab (BD Biosciences, cat. No. 551262). Subsequently, cells were stained with Streptavidin-APCCy7 1:200 (BD Biosciences, cat. No. 554063). DAPI (Sigma Aldrich, cat.no. D9542) was added at 1:10 000 to discriminate dead cells. Samples were acquired on Gallios flow cytometer (Beckman Coulter) and a LSRFortessa cell analyzer (BD Biosciences) and were analyzed with Kaluza analysis software (Beckman Coulter).

Cell culture

HPC-7 cells were maintained in IMDM medium supplemented with 10% FCS and 100 ng/ml of mSCF (PeProTech 250-03).

MS-5 cells were maintained in α-MEM medium supplemented with 10% FCS. To induce megakaryocyte differentiation, HPC-7 cells were cultured with or without MS-5 stromal cells in StemSpan H3000 medium, supplemented with 50 ng/ml of TPO (PeProTech 300-18) and 2U/mL of EPO (R&D 287-TC-500) up to 4 days. 10 μM of BRL37344 (β3-AR agonist; Sigma B169), 10 μM of clenbuterol (β2-AR agonist; Sigma C5423), 5 μM of H-89 (Protein kinase A inhibitor; Sigma B1427) and vehicle controls were added to the culture. To examine megakaryocyte differentiation, cells were incubated with anti-ckit-FITC ab (1:200, BD PharMingen 553354) and anti-CD41-PE ab (1:200, BD PharMingen 558040). DAPI was added at 1:10,000 to discriminate dead cells. Samples were analyzed by Gallios flow cytometer (Beckman Coulter).

Human umbilical cord blood CD34+ HSPCs were isolated using a CD34 Microbead kit (Miltenyi Biotec 130-046-702) following manufacturer's instructions, and were cultured with MS-5 stromal cells in Cellgro medium (CellGenix cat. no. 20802-0500), supplemented with 50 ng/ml of TPO (PeProTech 300-18) and 5 ng/ml of IL-1β (Cellgenix 1411-050) for 7-10 days, during which vehicle, 10 μM of BRL37344 (β3-AR agonist; Sigma B169) or 10 μM of clenbuterol (β2-AR agonist; Sigma C5423) were added to the culture. Conditioned medium was refreshed at day3 and day7 of the coculture. To examine megakaryocyte differentiation, cells were fixed and stained with anti-human CD61 antibody (Serotec MCA 728).

For mouse bone marrow long-term culture, femurs and tibias were flushed gently to obtain bone marrow cells. Cells were seeded and cultured in MyeloCult M5300 medium (StemCell Technologies, cat. no. 05350) supplemented with 10^{-6} M hydrocortisone (StemCell Technologies, cat. no. 07904) at 33°C for 14 days. Half of the medium was refreshed at day 7. At day 14 of culture, half of the medium was refreshed again and added with 50 ng/ml of TPO (PeProTech 300-18) for 4 days, during which vehicle, 10 μM of BRL37344 (β3-AR agonist; Sigma B169), 10 μM of clenbuterol (β2-AR agonist; Sigma C5423), or 100 μM of L-VINO (Insight Biotechnology 728944-69-2) were added to the culture. At day 18 of culture, cells were collected and subjected to flow cytometry analysis.

Immunofluorescence staining

Immunofluorescence staining of cryostat sections was performed as previously described (Isern et al., 2014), with minor modifications. Briefly, tissues were permeabilized with 0.1% Triton X-100 (Sigma) for 10 min at RT and blocked with TNB buffer (0.1 M Tris–HCl, pH 7.5, 0.15 M NaCl, 0.5% blocking reagent, Perkin Elmer) for 1 h at RT. Primary antibody incubations were conducted for 2 h at RT. Secondary antibody incubations were conducted for 1 h at RT. Repetitive washes were performed with PBS + 0.05% Triton X-100. Stained tissue sections were counterstained for 5 min with 5 μM DAPI and rinsed with PBS. Slides were mounted in Vectashield Hardset mounting medium (Vector Labs) and sealed with nail polish. Skull bones were fixed with 2% PFA for 2h in 4C, washed with PBS and cut through the sagittal suture. Each half was permeabilized with PBS-0.1% Triton with 20% goat serum (Thermo Scientific, cat. No. 9722) overnight at 4°C on the rocker. Endogenous biotin was blocked with the Avidin/biotin blocking kit (Vector Laboratories, cat. No. SP-2001) according to manufacturer's recommendation. Endogenous peroxidase was blocked with 0.4% peroxide (Sigma-Aldrich, cat. no. H1009) for 2h at RT. Subsequently, the skull bones were stained with rat anti-CD31 (BD Biosciences, cat. no. 551262) and rabbit anti-TH (Millipore, cat. no. AB152) in 0.1% Triton-20%goat serum-PBS (diluted 1:100 and 1:500 respectively) for 3 days, and washed over the next day with 0.05% Triton - PBS- at RT on a rocker. Samples were stained with secondary goat anti-rat-Alexa647 at 1:300 (Life Technologies, cat. no. A21247) and goat anti-rabbit biotinylated at 1:200 (Stratech Scientific, cat. no. 111-066-003) o/n at 4°C. Subsequently, the skull bones were treated with ABC amplification kit (Vector Labs) to detect Cy3-Tyrmide amplified signal from TH staining (Perkin Elmer, cat.no. SAT704A001EA). As a final step, the skull bones were stained with DAPI 1:1000 for 5 min. Images were acquired with Leica TCS SP5 confocal microscope with

10x objective. From each sample, 3 representative images were collected in different areas: frontal bone near bregma, central sinus of parietal bone and interparietal bone near lambda. The TH+ area was analyzed with ImageJ. For whole mount staining of long bones, tibia or femur BM thick sections were obtained with a cryostat and remaining OCT was removed by PBS washes. Samples were blocked and permeabilized in 0.1% Triton X-100 (Sigma) TNB (0.1 M Tris–HCl, pH7.5, 0.15 M NaCl, 0.5% blocking reagent, Perkin Elmer) overnight at 4°C. On the next day, samples were incubated with rabbit anti-TH (Merck AB152; 1:200), goat anti-CD31 (R&D AF3628 1:100), rat anti-EMCN (Insight Biotechnology sc-65495 1:100) or rat anti-CD31 (BD Biosciences Clone MEC13.3; 1:200) diluted in 0.1% Triton X-100 TNB overnight at 4°C. Samples were rinsed with 0.05% Triton X-100 PBS for 8 hours and incubated overnight with secondary antibodies including A546 Donkey anti-rabbit IgG (Invitrogen A10040; 1:200), Dylight650 donkey anti-rat IgG (Thermo Fisher SA5-10029; 1:300), Cy3-donkey anti-goat IgG (Jackson 705-165-147, 1:300) diluted in TNB. Samples were rinsed with 0.05% Triton X-100 PBS for 4 hours, washed with PBS and counterstained with DAPI (Sigma; 1:1000) to label cell nuclei. Images were acquired with Leica SP5 confocal microscope using 10x, 20x and 40x objectives and analyzed with ImageJ. At least 3 independent and randomly selected BM areas were imaged and analyzed per sample. To quantify neural fibers, total TH+area was normalized per total BM area labeled with DAPI. For quantification of sinusoidal vessel area, EMCN+ area > 150 μm distant to the bone surface was measured and normalized to BM area. Arteriolar (> 6 μm diameter) and capillary (< 6 μm diameter) CD31hiEMCN- vessel number was counted and normalized to BM area. To quantify transition zone vessels, CD31hiEMCNhi area < 150 μm close to the bone surface was quantified and normalized to the bone surface length.

For staining of megakaryocyte and megakaryocyte progenitor (Figures 3C–3F, 4J–4L, 5D–5F, 7G–7I, and S2A–S2D), BM femur thin sections were blocked with TNB for 1h at RT. Samples were then incubated with primary antibodies for 2 h at RT or overnight at 4°C, followed by secondary antibodies incubation for 1h at RT. Finally, samples were counterstained with DAPI to label cell nuclei. Repetitive washes were performed with PBS. At least 3 independent and randomly selected BM areas were imaged and analyzed by ImageJ. The following primary antibodies were used: CD41 (1:200, PE conjugated rat monoclonal antibody, BD PharMingen 558040), CD42d (1:100, APC conjugated armenian hamster monoclonal antibody, eBioscience 17-0421-80) and ckit (1:100, goat polyclonal antibody, R&D AF1356). The following secondary antibodies were used: Alexa Fluor 647 Goat-anti-armenian hamster IgG (1:200, Abcam ab173004) and Alexa Fluor 647 donkey-anti-goat IgG (1:200, Life Technologies A21447).

For staining of megakaryocyte localization relative to sinusoids (Figures 3I–3L and 5H–5J), BM femur thin sections were blocked with 0.1% Triton X-100 TNB for 1h at RT. Samples were then incubated with rat anti-EMCN (Insight Biotechnology sc-65495 1:100) antibody diluted in 0.1% Triton X-100 TNB overnight at 4°C. On the next day, samples were incubated with Alexa Fluor 647 goat-anti-rat IgG (1:200, Life Technologies, cat. no. A21247) secondary antibody for 1h at RT, followed by rat serum blocking (1:10 rat serum in 0.05% Triton X-100 TNB) for 10 min. Samples were incubated with third antibody, CD41 (1:200, PE conjugated rat monoclonal antibody, BD PharMingen 558040) for 2h at RT. Finally, samples were counterstained with DAPI to label cell nuclei. Repetitive washes were performed with PBS or 0.05% Triton X-100 PBS. At least 3 independent and randomly selected BM areas were imaged and analyzed by ImageJ.

For HSCs and megakaryocytes staining (Figures 7, S5, and S6), BM femur thin sections were blocked with TNB for 1h at RT and followed by Avidin/biotin blocking. Samples were then incubated with rat anti-CD150 (1:50, Biolegend Cat. No. 115902) and armenian hamster anti-CD42d (1:100, eBioscience 17-0421-80) primary antibodies overnight at 4°C. On the next day, samples were incubated with Alexa Fluor 555 goat-anti-rat IgG (1:200, Life Technologies A21434) and Alexa Fluor 647 Goat-anti-armenian hamster IgG (1:200, Abcam ab173004) secondary antibodies for 1h at RT, followed by rat serum blocking (1:10 rat serum in TNB) for 10 min. Samples were re-stained with biotinylated lineage antibodies (1:100, BD Biosciences) for 2h at RT. Finally, samples were incubated with Alexa Fluor 488 Streptavidin-conjugated antibody (1:200, Invitrogen S32354) for 1h at RT and counterstained with DAPI to label cell nuclei. At least 3 independent and randomly selected BM areas were imaged and analyzed by ImageJ.

For staining of human cord blood CD34+ HSPC co-culture (Figures 4D and 4E), cells were fixed with 1% PFA for 20 mins at RT. After PBS washes, cells were blocked with 10% donkey serum (Sigma D9663) diluted in PBS for 1h at RT, followed by 2h incubation of mouse-anti-human CD61 primary antibody (1:500, Serotec MCA 728). After PBS washes, cells were incubated with Alexa Fluor 546 donkey-anti-mouse IgG (1:200, Life Technologies, cat. no. A10036) secondary antibody for 1h at RT. Finally, samples were counterstained with DAPI to label cell nuclei. 10 independent and randomly selected fields in culture wells were imaged and analyzed by ImageJ.

RNA isolation and qPCR

RNA isolation was performed using Trizol Reagent (Sigma T9424). Reverse transcription was performed using the High-Capacity cDNA Reverse Transcription kit (Applied Biosystems 4368814), following the manufacturer's recommendations. qPCR was performed using the PowerUp SYBR Green Master Mix (Applied Biosystems A25742) and ABI PRISM® 7900HT Sequence Detection System. The expression level of each gene was determined by using the absolute quantification standard curve method. All values were normalized with *Gapdh* as endogenous housekeeping gene.

The following primers (mouse) were used:

NOS1-Fw: ACTGACACCCTGCACCTGAAGA
NOS1-Rv: GTGCGGACATCTTCTGACTTCC
NOS2-Fw: CAGCTGGGCTGTACAAACCTT
NOS2-Rv: CATTGGAAGTGAAGCGGTTCG
NOS3-Fw: CCTCGAGTAAAGAACTGGGAAGTG

NOS3-Rv: AACTTCCTTGGAAACACCAGGG
Gapdh-Fw: GCATGGCCTTCCGTGTTC
Gapdh-Rv: CTGCTTCACCACCTTCTTGAT

ELISA

MILLIPLEX MAP Mouse Cytokine/Chemokine Magnetic Bead Panel (MCYTOMAG-70K, Merck Millipore) was performed following the manufacture's protocol. Extracellular fluids of mouse long bones were collected from the supernatants following BM extraction and subjected to the mouse cytokine panel.

Measurement of nitrate concentration

NO(X) content was measured in freshly-thawed samples that had been kept at $-80^{\circ}C$ for less than 2 months using a nitric oxide analyzer (NOA) 280i (Siever, GE Healthcare) according to the manufacturer's instructions. Data were collected, processed and analyzed by using liquid software (Siever, GE Healthcare).

Statistical analyses

Statistical analyses and graphics were carried out with GraphPad Prism 5 software and Microsoft Excel.

DATA AND CODE AVAILABILITY

The published article includes all relevant datasets generated or analyzed during this study.

Cell Stem Cell, Volume *25*

Supplemental Information

Remodeling of Bone Marrow Hematopoietic Stem Cell Niches Promotes Myeloid Cell Expansion during Premature or Physiological Aging

Ya-Hsuan Ho, Raquel del Toro, José Rivera-Torres, Justyna Rak, Claudia Korn, Andrés García-García, David Macías, Cristina González-Gómez, Alberto del Monte, Monika Wittner, Amie K. Waller, Holly R. Foster, Carlos López-Otín, Randall S. Johnson, Claus Nerlov, Cedric Ghevaert, William Vainchenker, Fawzia Louache, Vicente Andrés, and Simón Méndez-Ferrer

Figure S1, Related to Figure 1 and Figure 2

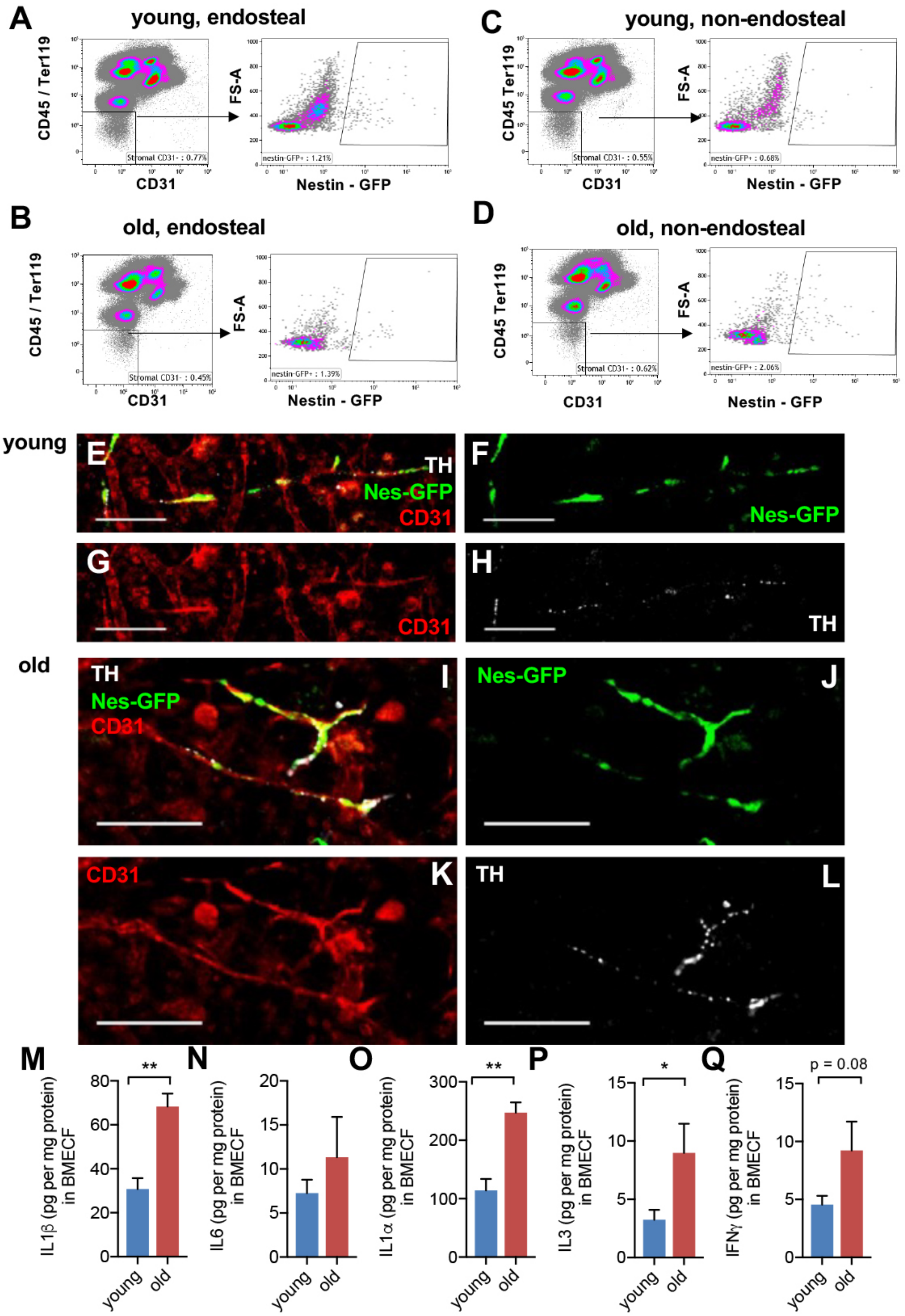

Figure S1, Related to Figure 1 and Figure 2. Reduction of endosteal niches and expansion of non-endosteal neurovascular niches correlates with increased myelopoietic cytokines during BM aging. (A-D) Representative flow cytometry diagrams from BM CD45$^-$ Ter119$^-$ Nes-GFP$^+$ cells isolated from (A, B) endosteal and (C, D) non-endosteal BM from (A, C) young and (B, D) old *Nes-gfp* mice. The frequencies of gated population are indicated. (E-L) Additional examples showing the immunofluorescence of tyrosine hydroxylase (TH)$^+$ sympathetic nerve fibers (white), CD31$^+$ endothelial cells (red), and GFP$^+$ cells (green) in the tibial BM of (E-H) young and (I-L) old *Nes-gfp* mice. Scale bar, 100 µm. (M-Q) Protein concentration of (M) IL1β, (N) IL6, (O) IL1α, (P) IL3 and (Q) IFNγ in non-endosteal BM extracellular fluid (BMECF) from young WT mice (n = 3) and old WT mice (n = 7). Data are means ± SEM. * $p < 0.05$; ** $p < 0.01$. Unpaired two-tailed t test.

Figure S2, Related to Figure 3

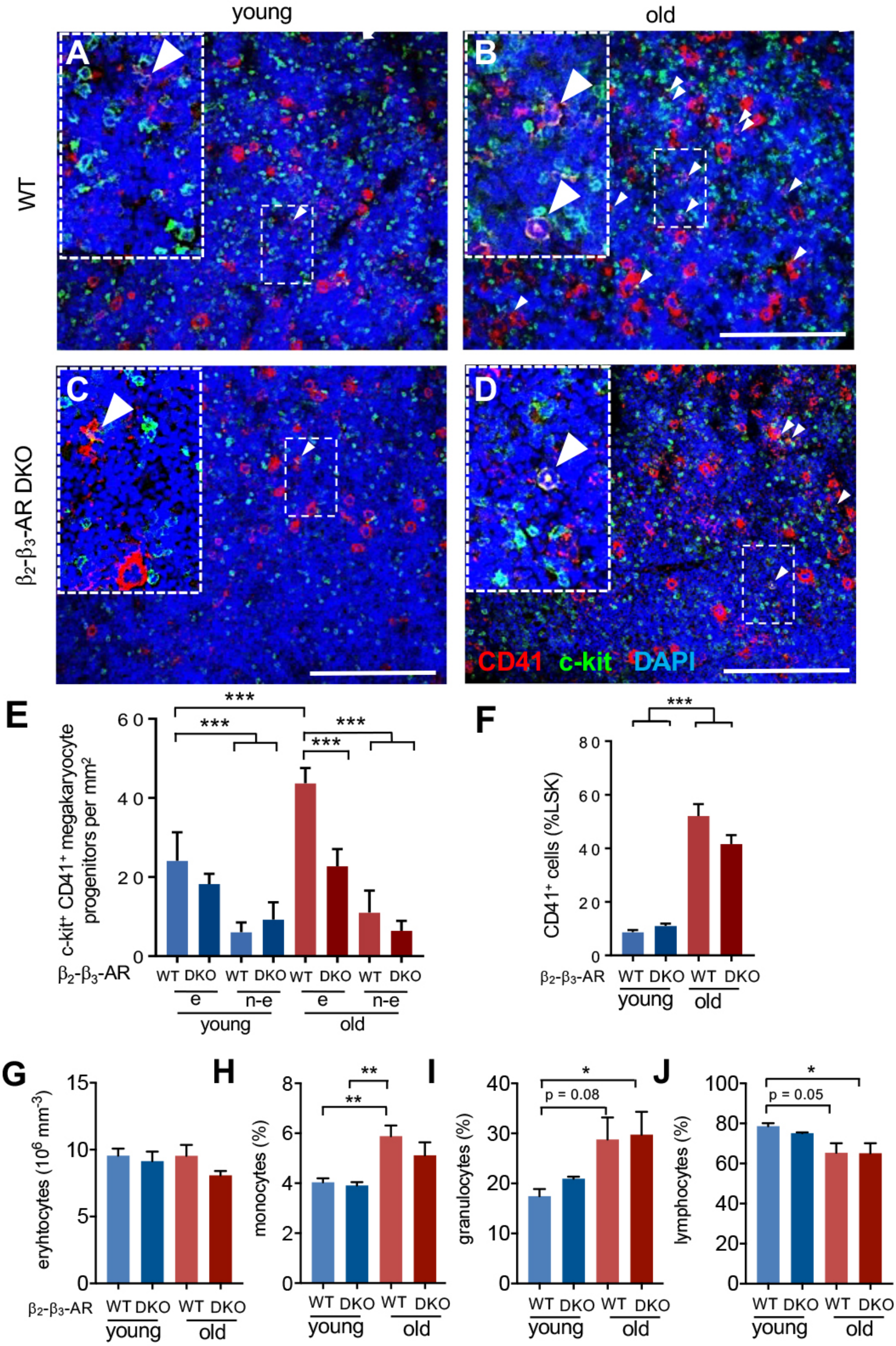

Figure S2, Related to Figure 3. β-adrenergic signals promote megakaryopoiesis during aging. (A-D) Representative immunofluorescence staining for CD41 (red) and c-kit (green) in femoral BM sections of WT or β_2-β_3-AR DKO mice of different age. Images were taken from the diaphysis regions; c-kit$^+$CD41$^+$ cells are depicted with white arrows. (E) c-kit$^+$CD41$^+$ cells per mm^2 (n = 4 young mice; n = 3 old mice) in endosteal (e) or non-endosteal (n-e) BM areas. Regions within 1/5 marrow width from the bone surface are defined as endosteal (both sides of the bone), while the remaining 3/5 marrow width in the central part are considered as non-endosteal. (F) Frequency of CD41$^+$ cells in non-endosteal BM Lin$^-$Sca-1$^+$c-kit$^+$ (LSK) HSPCs in endosteal BM from young WT mice (n = 7), young β_2-β_3-AR DKO mice (n = 7), old WT mice (n = 5), and old β_2-β_3-AR DKO mice (n = 4) measured by flow cytometry. (G-J) Peripheral blood counts from young WT mice (n = 8), young β_2-β_3-AR DKO mice (n = 8), old WT mice (n = 6) and old β_2-β_3-AR DKO mice (n = 7). (G) Erythrocytes. (H-J) Frequencies of (H) monocytes, (I) granulocytes and (J) lymphocytes among white blood cells. Data are means ± SEM. * $p < 0.05$; ** $p < 0.01$; *** $p < 0.001$ (One-way Anova followed by Bonferroni pairwise comparisons).

Figure S3, Related to Figure 4

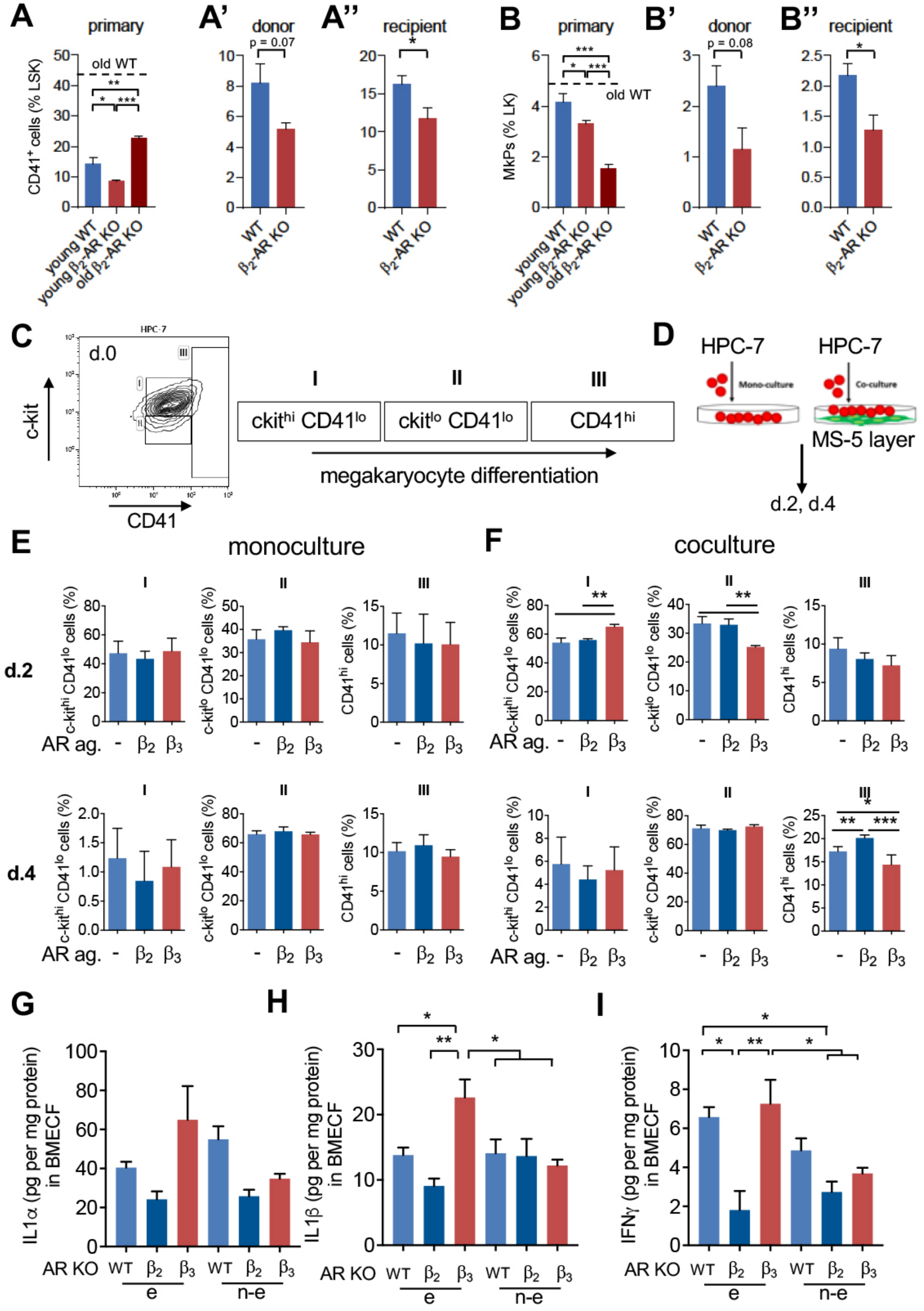

Figure S3, Related to Figure 4. β-ARs have opposite, stage-divergent and stromal-cell-dependent effects on megakaryocyte differentiation. (A-B) Frequency of (A, A', A'') $CD41^+$ myeloid or megakaryocyte progenitors within $lin^-sca\text{-}1^+c\text{-}kit^+$ (LSK) cells or (B, B', B'') $CD150^+CD41^+$ megakaryocyte progenitors (MkPs) within $lin^-c\text{-}kit^+$ (LK) cells in non-endosteal BM cells of the following mice: (A, B) young WT (A, n = 6; B, n = 3), young *Adrb2*$^{-/-}$ (n = 8) or old *Adrb2*$^{-/-}$ (n = 4) mice; (A', B') lethally-irradiated WT recipients of WT (n = 5) or *Adrb2*$^{-/-}$ (n = 4) BM cells; (A'', B'') lethally-irradiated WT (n = 5) or *Adrb2*$^{-/-}$ (n = 4) recipients of WT BM cells. (C) Representative flow cytometry diagram of HPC-7 cells before treatment with cytokines (day 0). Scheme showing the cell populations indicating progressive megakaryocyte commitment: I, $ckit^{hi}CD41^{lo}$ cells; II, $ckit^{lo}CD41^{lo}$ cells; III, $CD41^{hi}$ cells. Megakaryocyte differentiation trajectory flows from populations I → II → III. (D) Scheme showing the experimental design. HPC-7 cells were cultured alone or co-cultured with MS-5 stromal cells in megakaryocyte differentiation medium. (E-F) Quantification of cell populations I, II and III after 2 or 4 days of treatment with vehicle, β_2-AR agonist or β_3-AR agonist in (E) monoculture or (F) coculture with MS-5 stromal layers (n = 4). (G-I) Concentration of (G) IL1α, (H) IL1β and (I) IFNγ in endosteal (e) or non-endosteal (n-e) BM extracellular fluid (BMECF) from adult WT mice (n = 6), *Adrb2*$^{-/-}$ mice (n = 4), or *Adrb3*$^{-/-}$ mice (n = 7). Data are means ± SEM. * $p < 0.05$; ** $p < 0.01$; *** $p < 0.001$. (A, B, E-I) One-way Anova followed by Bonferroni pairwise comparisons. (A', A'', B', B'') Unpaired two-tailed t test.

Figure S4, Related to Figure 5

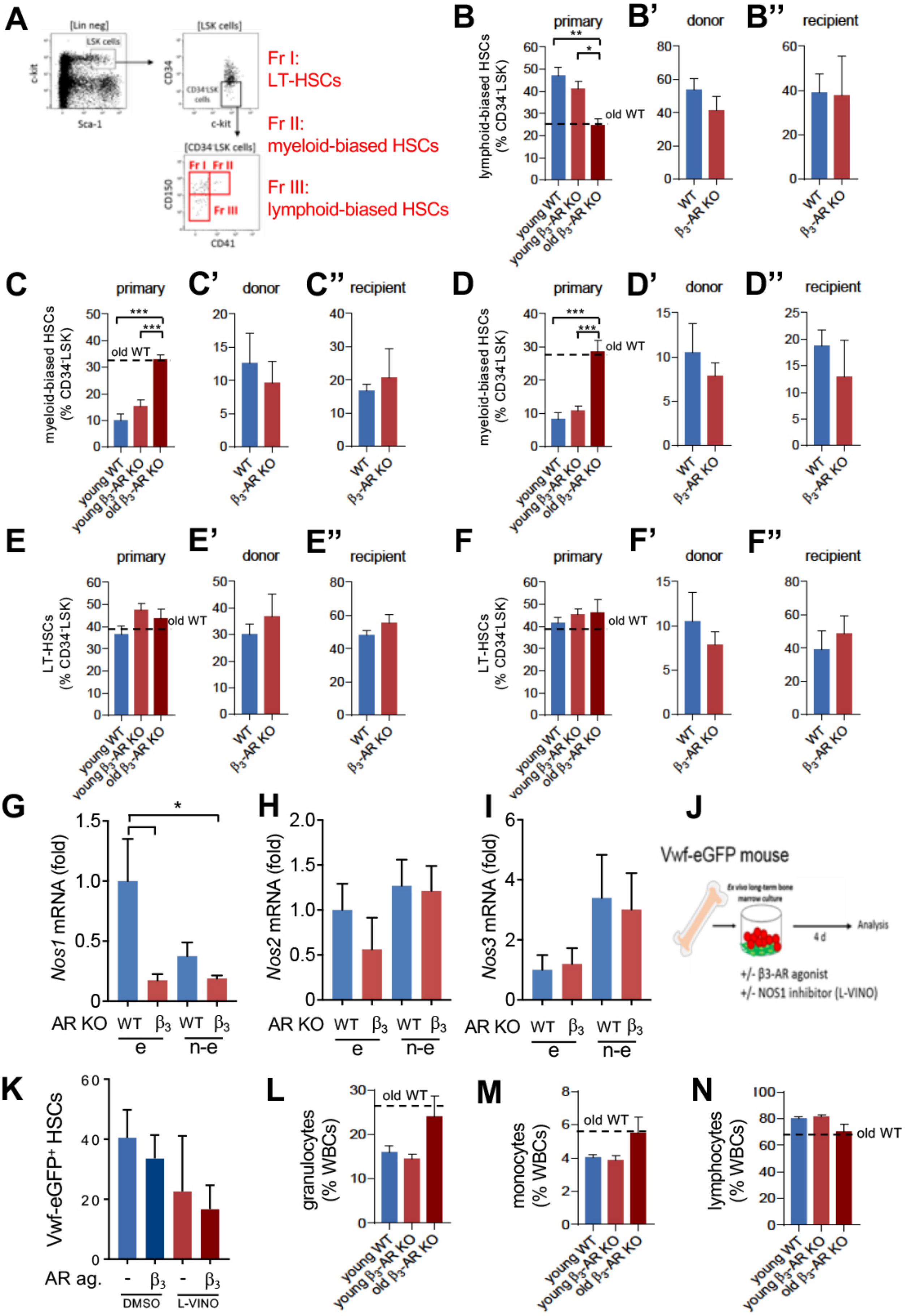

Figure S4, Related to Figure 5. β3-AR regulates HSC lineage bias partially through Nos1-dependent NO production. (A) Representative flow cytometry diagram of HSC populations based on CD41 and CD150 expression. (B) Frequency of $CD150^{lo/-}CD41^{-}$ lymphoid-biased HSCs in non-endosteal BM $CD34^{-}LSK$ cells. (C-F) Frequency of (C-D) $CD150^{+}CD41^{+}$ myeloid-biased HSCs or (E-F) $CD150^{+}CD41^{-}$ LT-HSCs in (C, E) endosteal or (D, F) non-endosteal BM $CD34^{-}LSK$ cells from the following mice: (B, C, D, E, F) young WT (n = 7), young *Adrb3*$^{-/-}$ (n = 7) or old *Adrb3*$^{-/-}$ (n = 5) mice; (B', C', D', E', F') lethally-irradiated WT recipients of WT (n = 5) or *Adrb3*$^{-/-}$ (n = 5) BM cells; (B'', C'', D'', E'', F'') lethally-irradiated WT (n = 4) or *Adrb3*$^{-/-}$ (n = 3) recipients of WT BM cells. (G-I) mRNA levels (fold) of (G) *Nos1* (H) *Nos2* and (I) *Nos3* in the endosteal (e) or non-endosteal (n-e) BM of adult WT mice (n = 6) and β_3-AR KO mice (n = 7). (J) Scheme of long-term BM culture from *Vwf-eGFP* mice. (K) Number of $lin^{-}sca\text{-}1^{+}c\text{-}kit^{+}CD34^{-}CD48^{-}CD150^{+}Vwf\text{-}eGFP^{+}$ platelet-biased HSCs in primary BM culture from *Vwf-eGFP* mice treated with β_3-AR agonist (BRL37344, 10 μM), *Nos1* inhibitor (L-VINO, 100 μM) or vehicle for 4 days (n = 8). (L-N) Frequency of (L) granulocytes, (M) monocytes and (N) lymphocytes in white blood cells (WBCs) in young WT (L, N, n = 10; M, n = 8), young *Adrb3*$^{-/-}$ (n = 4) or old *Adrb3*$^{-/-}$ (n = 6) mice. Data are means ± SEM. * $p < 0.05$; ** $p < 0.01$. (B', B''-F', F'') Unpaired two-tailed *t* test. (B-F, G-N) One-way Anova followed by Bonferroni pairwise comparisons.

Figure S5, Related to Figure 6

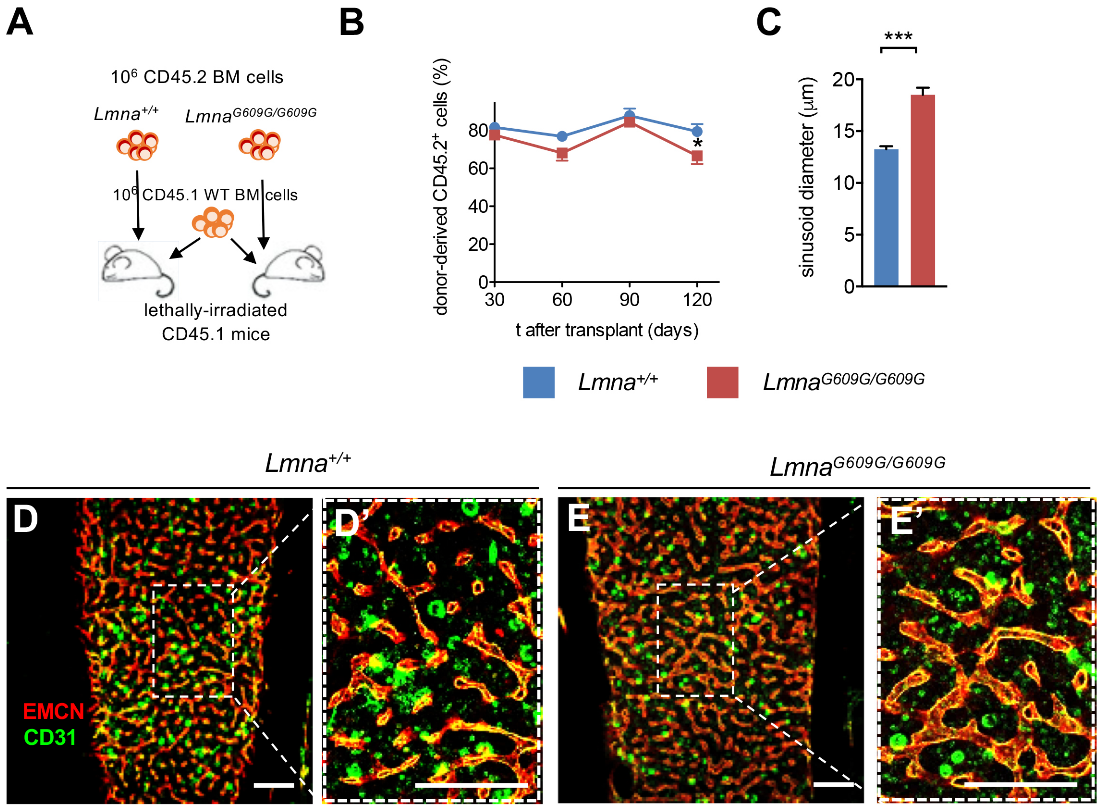

Figure S5, Related to Figure 6. Characterization of hematopoiesis and BM vasculature in HGPS mice. (A) Scheme of competitive BM transplantation. (B) Frequency of CD45.2$^+$ donor-derived hematopoietic cells in peripheral blood of recipient mice 30, 60, 90 and 120 days after lethal irradiation and transplantation with 10^6 BM cells from WT (blue) or *Lmna*$^{G609G/G609G}$ (red) mice, together with 10^6 competitor BM cells from CD45.1 C57BL/6 mice. (C) Quantification of sinusoidal (EMCN$^+$ vasculature) diameter in each group of mice. (D, E) Representative immunofluorescence of CD31 (green) and EMCN (red) in thick femoral BM sections from adult WT mice (*Lmna*$^{+/+}$; n = 7) and *Lmna*$^{G609G/G609G}$ mice (n = 7). Scale bar, 200 µm. (D', E') Insets of D and E. Scale bar, 100 µm. (B-C) Data are means ± SEM. * $p < 0.05$; *** $p < 0.001$ (unpaired two-tailed *t* test).

Figure S6, Related to Figure 6 and Figure 7

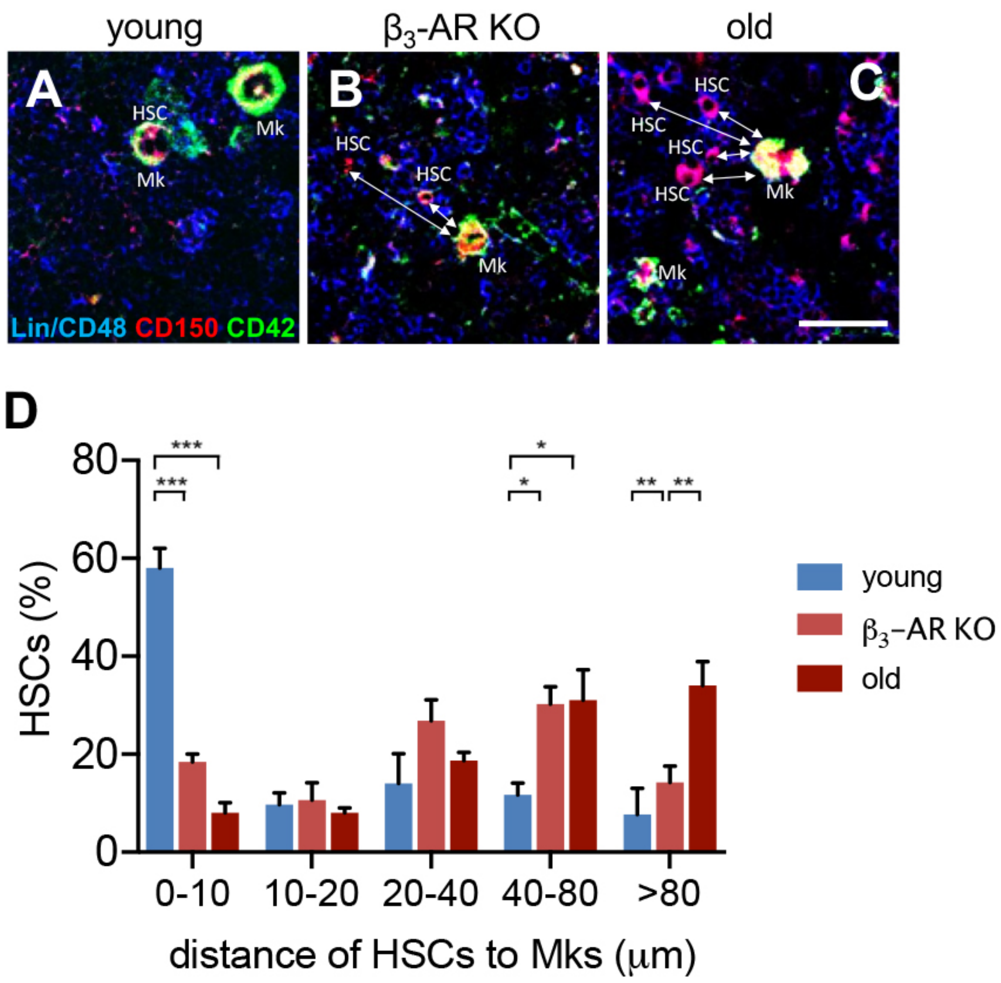

Figure S6, Related to Figure 7. Decreased HSC-megakaryocyte interactions in adult β3-AR KO mice resemble aged WT mice. (A-D) Representative immunofluorescence (A-C) and distribution (D) of BM CD150$^+$ (red) HSCs (negative for mature hematopoietic lineage markers, blue) adjacent (asterisks) or nonadjacent (arrows) to CD42$^+$ (green) megakaryocytes in femoral BM sections of young WT mice (n = 3), adult *Adrb3*$^{-/-}$ mice (n = 5) and old WT mice (n = 3). Scale bar, 50 µm. Data are means ± SEM. * p < 0.05; ** p < 0.01; *** p < 0.001 (Two-way Anova and Bonferroni pairwise comparisons).

Figure S7, Related to Figure 6

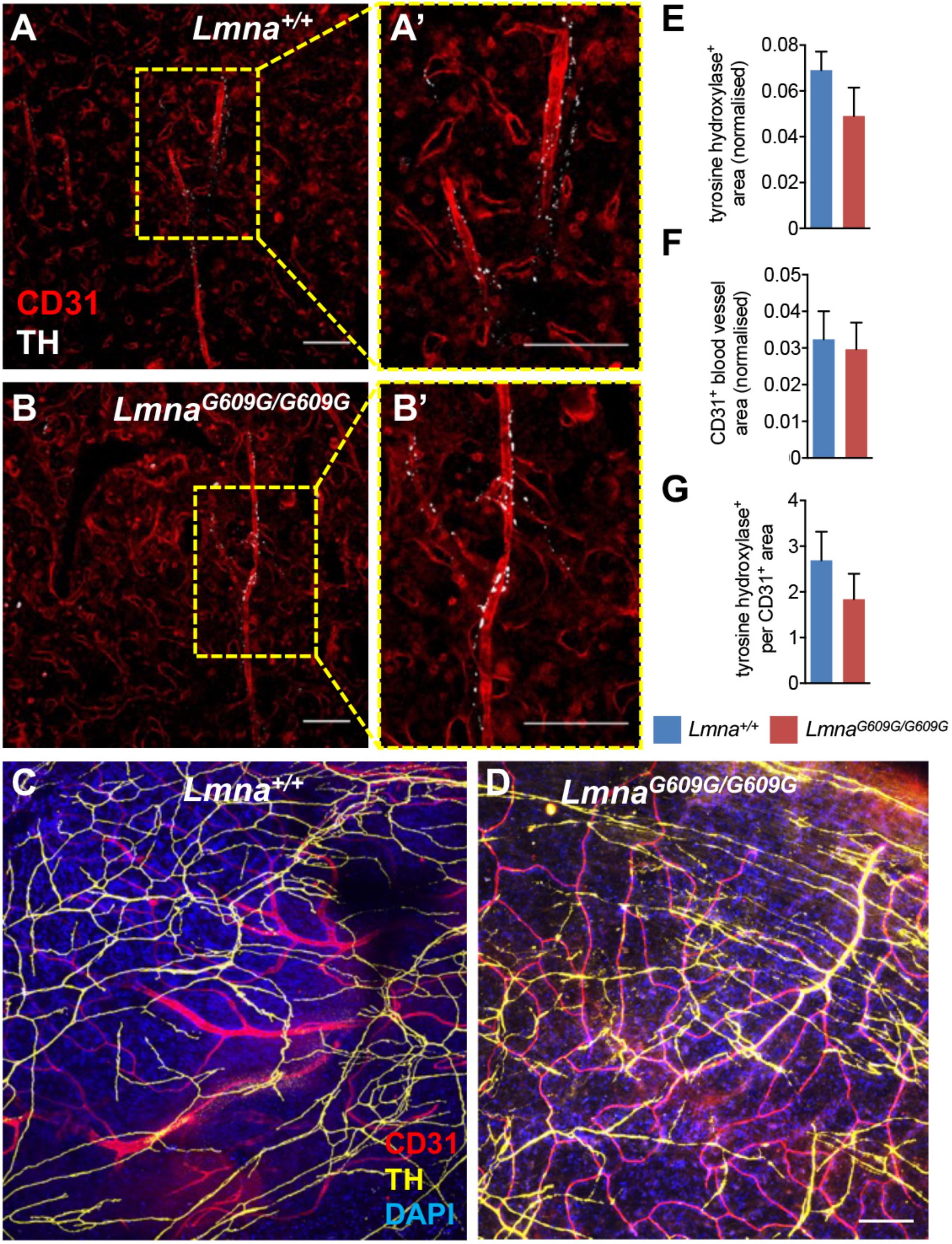

Figure S7, Related to Figure 6 and 7. BM noradrenergic innervation is not reduced in a mouse model of premature aging. (A-D) Whole-mount immunofluorescence staining of tyrosine hydroxylase $(TH)^+$ sympathetic nerve fibers (A, B, white; C, D, yellow) and $CD31^+$ endothelial cells (red) in the femoral BM (A, B) and skull (C, D) of (A, C) adult WT mice ($Lmna^{+/+}$; n = 6) and (B, D) $Lmna^{G609G/G609G}$ mice (n = 7). A', B', insets of A and B. Scale bar, 100 µm (A, B), 200 µm (C, D). (E) Quantification of the area covered by TH^+ fibers in the skull. (F) Quantification of the area covered by $CD31^+$ endothelial cells in the skull. (G) Ratio of the area of TH^+ fibers to $CD31^+$ endothelial cells in the skull. Data are means ± SEM.
